# Regulatory Role of Non-Coding RNAs on Immune Responses During Sepsis

**DOI:** 10.3389/fimmu.2021.798713

**Published:** 2021-12-09

**Authors:** Soudeh Ghafouri-Fard, Tayyebeh Khoshbakht, Bashdar Mahmud Hussen, Mohammad Taheri, Normohammad Arefian

**Affiliations:** ^1^ Department of Medical Genetics, School of Medicine, Shahid Beheshti University of Medical Sciences, Tehran, Iran; ^2^ Phytochemistry Research Center, Shahid Beheshti University of Medical Sciences, Tehran, Iran; ^3^ Department of Pharmacognosy, College of Pharmacy, Hawler Medical University, Erbil, Iraq; ^4^ Center of Research and Strategic Studies, Lebanese French University, Erbil, Iraq; ^5^ Institute of Human Genetics, Jena University Hospital, Jena, Germany; ^6^ Skull Base Research Center, Loghman Hakim Hospital, Shahid Beheshti University of Medical Sciences, Tehran, Iran

**Keywords:** lncRNA, miRNA, sepsis, expression, biomarker

## Abstract

Sepsis is resulted from a systemic inflammatory response to bacterial, viral, or fungal agents. The induced inflammatory response by these microorganisms can lead to multiple organ system failure with devastating consequences. Recent studies have shown altered expressions of several non-coding RNAs such as long non-coding RNAs (lncRNAs), microRNAs (miRNAs) and circular RNAs (circRNAs) during sepsis. These transcripts have also been found to participate in the pathogenesis of multiple organ system failure through different mechanisms. NEAT1, MALAT1, THRIL, XIST, MIAT and TUG1 are among lncRNAs that participate in the pathoetiology of sepsis-related complications. miR-21, miR-155, miR-15a-5p, miR-494-3p, miR-218, miR-122, miR-208a-5p, miR-328 and miR-218 are examples of miRNAs participating in these complications. Finally, tens of circRNAs such as circC3P1, hsa_circRNA_104484, hsa_circRNA_104670 and circVMA21 and circ-PRKCI have been found to affect pathogenesis of sepsis. In the current review, we describe the role of these three classes of noncoding RNAs in the pathoetiology of sepsis-related complications.

## Introduction

Sepsis is a systemic inflammatory response to different infections, namely bacterial, viral, or fungal agents. This condition is the principal source of mortality in intensive care units (1). These infectious microorganisms can stimulate inflammatory reactions through induction of cytokines release. These reactions lead to multiple organ system failure. Other factors that contribute in this devastating condition during sepsis are systemic hypotension and abnormal perfusion of the microcirculatory system (2). No specific treatment modality has been suggested for prevention of multiple organ system failure during sepsis (2). Thus, identification of sepsis-related changes at cellular and biochemical levels is important. Currently, there is no effective pharmacological therapy for sepsis. Thus, early diagnosis, resuscitation and instant administration of suitable antibiotics are essential steps in decreasing the burden of this condition {Thompson, 2019 #562}.

Lipopolysaccharide (LPS) as the main constituent of the cell wall of Gram-negative bacteria has been found to stimulate apoptotic pathways in tubular epithelial cells of kidney (3). Moreover, it can prompt acute inflammatory responses through activation of NF-κB during the course of acute kidney injury (4). This molecular pathway is an important axis in mediation of immune-related organ damage.

Recent studies have shown altered expressions of several non-coding RNAs such as long non-coding RNAs (lncRNAs), microRNAs (miRNAs) and circular RNAs (circRNAs) during sepsis. These transcripts have also been found to participate in the pathogenesis of multiple organ system failure through different mechanisms. In the current review, we describe the role of these three classes of noncoding RNAs in the pathoetiology of sepsis-related complications.

## LncRNAs and Sepsis

LncRNAs are transcripts with sizes larger than 200 nucleotides. These transcripts regulate gene expression through modulation of chromatin configuration, regulation of splicing events, serving as decoys for other transcripts and making structures for recruitment of regulatory proteins (5). These transcripts participate in the regulation of immune reactions and pathoetiology of several immune-related disorders (6).

Experiments in animal model of acute lung injury have shown down-regulation of TUG1 and induction of apoptosis and inflammation. Up-regulation of TUG1 in these animals could ameliorate sepsis-associated lung injury, apoptosis and inflammatory reactions. TUG1 could also protect lung microvascular endothelial cells from deteriorative effects of LPS. In fact, TUG1 inhibits cell apoptosis and inflammatory reactions in LPS-stimulated microvascular endothelial cells through sponging miR-34b-5p and releasing GAB1 from its inhibitory effects. Cumulatively, TUG1 ameliorates sepsis-associated inflammation and apoptosis through miR-34b-5p/GAB1 axis (7). Another study has demonstrated down-regulation of TUG1 while up-regulation of miR-223 in the plasma samples of sepsis patients. They have also reported a negative correlation between expressions of TUG1 and miR-223 in sepsis patients. Besides, expression levels of TUG1 have been negatively correlated with respiratory infection, serum creatinine, white blood cell, C-reactive protein, APACHE II score, and SOFA score. Based on these results, TUG1 has been suggested as a biomarker for prediction of course and prognosis of sepsis (8). TUG1 has also been shown to interact with miR-27a. Over-expression of TUG1 has resulted in down-regulation of TNF-α, while up-regulation of miR-27a has enhanced expression of TNF-α in cardiomyocytes. TNF-α and miR-27a up-regulation could enhance LPS-induced apoptosis of cardiomyocytes. On the other hand, TUG1 up-regulation has exerted opposite effects (9).

**Figure 1:**
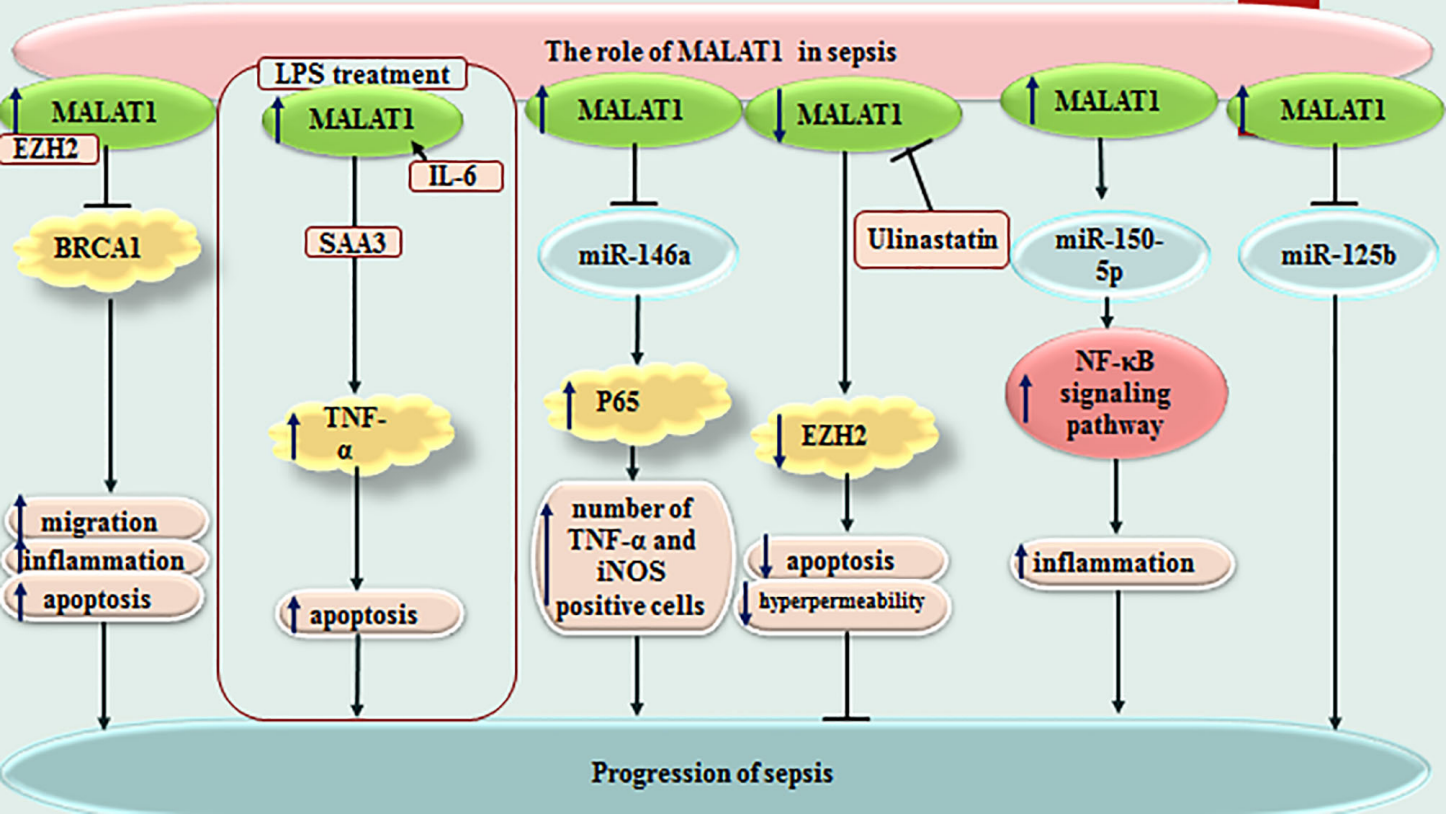
Function of MALAT1 in sepsis-related events.

MALAT1 is another lncRNA that affects immune responses of rats with LPS-induced sepsis through influencing the miR-146a/NF-κB P65 axis (10). Moreover, MALAT1 could increase apoptosis skeletal muscle cells and sepsis-associated immune responses through down-regulating BRCA1 levels *via* recruitment of EZH2 (11). The miR-150-5p/NF-κB axis is another axis that mediates the effects of MALAT1 in sepsis-associated cardiac inflammation (12). In addition, the protective effects of Ulinastatin against LPS-associated dysfunction of heart microvascular endothelial cells have been shown to be exerted through down-regulation of MALAT1 (13). Most notably, MALAT1/miR-125a axis has been shown to discriminate sepsis patients based on their severity of diseases, organ damage, levels of inflammatory responses and mortality (14). **Figure 1** depicts function of MALAT1 in sepsis-related events.

**Figure 2:**
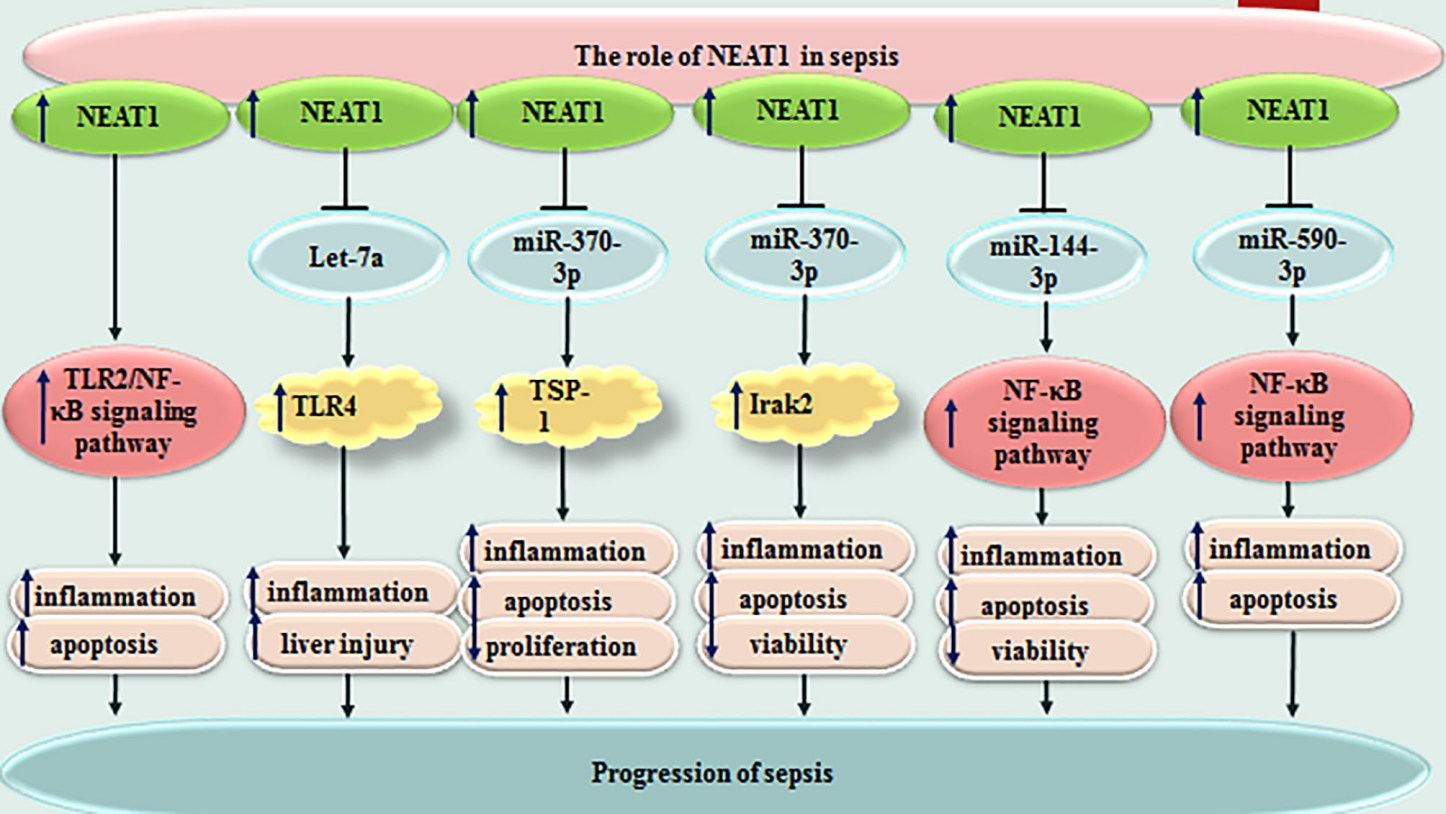
Function of NEAT1 in sepsis-related events. Several other lncRNAs have also been found to influence course of sepsis through modulation of immune responses (**Table 1**).

NEAT1 is another lncRNA whose participation in the pathophysiology of sepsis has been vastly investigated. This lncRNA could promote inflammatory responses and aggravate sepsis-associated hepatic damage through the Let-7a/TLR4 axis (15). Moreover, NEAT1 can accelerate progression of sepsis *via* miR-370-3p/TSP-1 axis (16). This lncRNA could also promote LPS-induced inflammatory responses in macrophages through regulation of miR-17-5p/TLR4 axis (17). NEAT1 silencing could suppress immune responses during sepsis through miR‐125/MCEMP1 axis (18). **Figure 2** shows the function of NEAT1 in sepsis-related events. Several other lncRNAs have also been found to influence course of sepsis through modulation of immune responses (**Table 1**).

**Table 1:** LncRNAs and Sepsis.

lncRNA	Expression Pattern	Clinical Samples/ Animal Model	Assessed Cell Lines	Targets / Regulators	Signaling Pathways	Description	Reference
TUG1	↓	35 ARDS patients and 68 HCs, male C57BL/6 mice	PMVECs	↑ miR-34b-5p, GAB1 ↓	_	TUG1 reduces sepsis-induced pulmonary injury, apoptosis and inflammation in ALI.	(7)
TUG1	↓	122 patients with sepsis and 122 HCs	_	↑ miR-223	_	Low levels of TUG1 was correlated with respiratory infection. TUG1 expression was negatively associated with Scr, WBC, SOFA score, and CRP levels and 28‐day deaths, but positively associated with albumin levels.	(8)
TUG1	↓	_	HUVECs	↑ miR-27a-3p, ↓ SLIT2	_	Up-regulation of TUG1 reduced apoptosis, autophagy, and inflammatory response.	(19)
TUG1	↓	70 patients with sepsis and 70 HCs	AC16	miR-27a, ↑ TNF-α	_	Up-regulation of TUG1 reduced apoptosis.	(9)
MALAT1	↑	rats with and without LPS-induced sepsis	U937	↓ miR-146a, ↑ P65	↑ NF-κB signaling pathway	Downregulation of MALAT1 decreased the number of TNF-α and iNOS positive cells.	(10)
MALAT1	↑	BALB/c male mice	HSMKMC 3500	↓ BRCA1, EZH2	_	Downregulation of MALAT1 reduced inflammatory responses, neutrophil migration, skeletal muscle cell apoptosis, and AKT-1 phosphorylation.	(11)
MALAT1	↑	_	H9c2	↓ miR-150-5p,	↑ NF-κB signaling pathway	Downregulation of MALAT1 reduced inflammatory response and downregulated NF-κB signaling pathway.	(12)
MALAT1	↑	male SD rats	CMVECs	↑ EZH2	_	MALAT1 significantly inhibited levels of EZH2 target genes, DAB2IP and Brachyury. Up-regulation of CRNDE increased permeability and apoptosis. Ulinastatin suppressed levels of MALAT1 and EZH2.	(13)
MALAT1	↑	196 patients with sepsis and 196 HCs,	_	↓ miR‐125a	_	MALAT1 expression was positively correlated with APACHE II score, SOFA score, serum creatinine, CRP, TNF‐α, IL‐1β, IL‐6, 28‐day deaths, and negatively with albumin.	(14)
MALAT1	↑	sepsis mice	_	↓ miR-23a, ↑ MCEMP1	_	Downregulation of MALAT1 suppressed expression of MPO, IL-6, IL-10, TNF-α, and IL-1β, and reduced inflammation.	(20)
MALAT1	↑	male C57 mice		↑ p38	↑ p38 MAPK/p65 NF-κB signaling pathway	Downregulation of MALAT1 reduced MPO and inflammatory responses.	(21)
MALAT1	↑	_	a lung injury inflammatory cell model	↓ miR-149, ↑ MyD88	↑ NF-κB pathway	Downregulation of MALAT1 reduced the levels of MyD88, TNF-α, IL-1β, and IL-6, and prevented the NF-κB pathway.	(22)
MALAT1	↑	CLP-induced septic mice	HUVECs, PAECs	↓ miR-150	↑ NF-κB pathway	Downregulation of MALAT1 reduced apoptosis, ER stress and inflammation.	(23)
MALAT1	↑ in ARDS group	152 patients with sepsis (41 ARDS and 111 Non-ARDS patients)	_	_	_	MALAT1 expression was association with APACHE II score, SOFA score, inflammatory factors levels, and high mortality.	(24)
MALAT1	↑	GEO dataset (GSE3140), male C57B6/L mice	HL-1	↑ IL-6, ↑ ↑ TNF-α, SAA3	_	Downregulation of MALAT1 Protected Cardiomyocytes from LPS-induced Apoptosis.	(25)
MALAT1	↑	190 patients with sepsis and 190 HCs	_	↓ miR‐125b	_	MALAT1 expression was associated with Scr, WBC, CRP, PCT, TNF‐α, IL‐8, IL‐17, APACHE II score, SOFA score, and 28‐day deaths.	(26)
MALAT1	↑	120 patients with sepsis and 60 HCs	_	_	_	Expression of MALAT1 was found to be an independent risk factor for sepsis, poor prognosis and septic shock.	(27)
MALAT1	↑	female C57BL/6 mice	THP-1	↓ miR-214, ↑ TLR5	_	Downregulation of MALAT1 attenuated the burn injury and post-burn sepsis-induced inflammatory reaction.	(28)
KCNQ1OT1	↓	male SD rats	H9c2	↑ miR-192-5p, ↓ XIAP	_	Up-regulation of KCNQ1OT1 ameliorated proliferation and impeded apoptosis in sepsis-induced myocardial injury.	(29)
CYTOR	↓	male SD rats	H9c2	↑ miR-24, ↓ XIAP	_	Up-regulation of CYTOR ameliorated viability and inhibited apoptosis in sepsis-induced myocardial injury.	(30)
lncRNA-5657	↑	15 patients with sepsis-induced ARDS and 15 non-septic and non-ARDS patients, SD rats	NR8383	↑ Spns2	_	Downregulation of lncRNA-5657 7 prevented sepsis-induced lung injury and LPS-induced inflammation.	(31)
RMRP	↓	male C57BL/6 mice	HL-1	↑ miR-1-5p, ↓ HSPA4	↑ NF-κB Pathway	Up-regulation of RMRP reduced LPS-induced damage, apoptosis and mitochondrial damage and LPS-induced sepsis.	(32)
NEAT1	↑	15 patients with sepsis-induced liver injury and 15 HCs	Kupffer, Raw264.7	↓ Let-7a, ↑ TLR4	_	Downregulation of NEAT1 reduced expression of inflammatory factors in sepsis-induced liver injury.	(15)
NEAT1	↑	25 Sepsis patients and 25 HCs	RAW 264.7	↓ miR-370-3p, ↑ TSP-1	_	Downregulation of NEAT1 prevented LPS-mediated inflammation and apoptosis and ameliorated proliferation.	(16)
NEAT1	↑	male pathogen-free C57BL/6 mice	_	↓ miR-125, ↑ MCEMP1	_	Downregulation of NEAT1 suppressed inflammation and T lymphocyte apoptosis.	(18)
NEAT1	↑	68 patients with sepsis and 32 HCs	THP-1 macrophages	↓ miR-17-5p, ↑ TLR4	_	Downregulation of NEAT1 prevented LPS-induced inflammatory responses in macrophages.	(17)
NEAT1	↑	mouse with sepsis-induced lung injury	_	↓ miR-16-5p, ↑ BRD4	_	Downregulation of NEAT1 inhibited inflammation, apoptosis, pulmonary edema, MPO activity, pathological changes, promoted viability.	(33)
NEAT1	↑	male C57 mice	_	_	↑ TLR2/NF-κB signaling pathway	Downregulation of NEAT1 reduced LPS-induced myocardial pathological injury, apoptosis, oxidative stress, inflammatory responses.	(34)
NEAT1	↑	male C57BL/6 mice	A549	_	↑ HMGB1/RAGE signaling	Downregulation of NEAT1 increased viability attenuated LPS-induced apoptosis and suppressed inflammation.	(35)
NEAT1	↑	30 patients with sepsis and 30 HCs	HK-2	↓ let-7b-5p, TRAF6	_	Downregulation of NEAT1 increased proliferation and inhibited apoptosis and inflammation.	(36)
NEAT1	↑	_	RAW264.7	↓ miR-125a-5p, ↑ TRAF6, ↑ P-TAK1	_	Downregulation of NEAT1 decreased inflammation by promoting macrophage M2 polarization.	(37)
NEAT1	↑	_patients with sepsis	HK2	↓ miR-93-5p, ↑ TXNIP	_	Downregulation of NEAT1 inhibited apoptosis, inflammation and oxidative stress.	(38)
NEAT1	↑	_ sepsis tissues and ANCTs	AW 264.7 and HL-1	↓ miR-370-3p, ↑ Irak2	_	Downregulation of NEAT1 ameliorated viability, prevented apoptosis and the expression of inflammatory cytokines.	(39)
NEAT1	↑	_	HL-1	↓ miR-144-3p	NF-κB signaling pathway	Downregulation of NEAT1 ameliorated viability, prevented apoptosis and inflammatory response in LPS-induced myocardial cell injury.	(40)
NEAT1	↑	152 patients with sepsis and 150	_	_	_	Up-regulation of NEAT1 was positively associated with Acute Physiology and Chronic Health Evaluation II score, inflammatory responses, while negatively associated with IL-10.	(41)
NEAT1	↑	C57BL/6 mice	WI-38	↓ miR-944, ↑ TRIM37	_	Downregulation of NEAT1 inhibited inflammatory responses and apoptosis. Overexpression of TRIM37 rescued influence of downregulation of NEAT1 on cell s.	(42)
NEAT1	↑	59 patients with sepsis, 52 patients with noninfectious SIRS, and 56 HCs	PBMCs	_	_	Levels of NEAT1 could be considered as a good predictor for the diagnosis of sepsis.	(43)
NEAT1	↑	127 patients with sepsis and 50 HCs	_	↑ Th1, ↑ Th17	_	Overexpression of NEAT1 was associated with chronic health evaluation II score, CRP level, acute physiology, and SOFA score.	(44)
NEAT1	↑	male C57BL/6 mice	RAW264.7	↓ miR495-3p, ↑STAT3, ↓ miR-211	↑ PI3K/AKT signaling	Overexpression of NEAT1 was associated with inflammatory responses.	(45)
NEAT1	↑	102 patients with sepsis and 100 HCs	_	↓ miR‐125a	_	High levels of NEAT1 was associated with SOFA score, APACHE II score, 28‐day deaths, and high ARDS risk.	(46)
NEAT1	↑	Septic Mice	_	↑ NF-κB	_	Downregulation of NEAT1 increased activity of nerve cells and reduced apoptosis.	(47)
NEAT1	↑	82 patients with sepsis and 82 HCs	_	↓ miR-124	_	NEAT1 showed a good predictive value for increased sepsis risk. NEAT1 expression was positively associated with disease severity, CRP, PCT, TNF-α, and IL-1β, 28-day deaths.	(48)
NEAT1	↑	18 patients with sepsis-induced AKI and 18 HCs	HK-2	↓ miR-22-3p	↑ NF-κB pathway	Downregulation of NEAT1 reduced levels of autophagy factors and inflammatory responses.	(49)
NEAT1	↑	_	RAW264.7	↓ miR-31-5p, ↑ POU2F1	_	Downregulation of NEAT1 reduced inflammatory response and apoptosis, and increased proliferation.	(50)
NEAT1	↑	22 patients with sepsis and 22 HCs,	H9c2	↓ miR-590-3p	NF-κB signaling pathway	Downregulation of NEAT1 reduced apoptosis and inflammatory responses in LPS-induced sepsis.	(51)
H19	↓	69 patients with sepsis and HCs, male BALB/c mice	_	↑ miR-874, ↓ AQP1	_	Downregulation of H19 contributed to inflammatory responses. Up-regulation of H19 ameliorated the impairment of sepsis companied myocardial dysfunction.	(52)
H19	↓	_	H9C2	↑ miR-93-5p, ↓ SORBS2	_	Up-regulation of H19 suppressed inflammatory responses in sepsis-induced myocardial injury.	(53)
H19	↓	104 patients with sepsis, and 92 HCs	_	_	_	Expression of H19 was negatively associated with 28-day deaths and inflammatory response markers.	(54)
CASC9	↓	rats	HSAECs	↑ miR-195-5p, ↓ PDK4	_	Up-regulation of CASC9 promoted viability in sepsis-induced ALI.	(55)
LUADT1	↓	60 patients with sepsis and 60 HCs	HCAECs	miR-195, ↓ Pim-1	_	Up-regulation of LUADT1 reduced apoptosis.	(56)
MIAT	↑	male SD rats	NRK-52E	↓ miR-29a	_	Up-regulation of MIAT promoted apoptosis in sepsis-related kidney injury.	(57)
MIAT	↑	male BALB/c mice	HL-1	↓ miR-330-5p, ↑ TRAF6	↑ NF-κB signaling	Downregulation of MIAT restrained inflammation and oxidative stress in Sepsis-Induced Cardiac Injury.	(58)
THRIL	↑	66 patients with sepsis and 66 HCs	HBEpCs	↓ miR-19a, ↑ TNF-α	_	Up-regulation of THRIL promoted apoptosis.	(59)
THRIL	↑	C57BL/6 mice	MPVECs	↓ miR-424, ↑ ROCK2	_	Downregulation of THRIL prevented inflammatory responses, and apoptosis in septic-induced acute lung injury.	(60)
THRIL	↑ in ARDS group	32 sepsis patients with ARDS and 77 without ARDS	_	_	_	THRIL independently predicted increased risk of ARDS. THRIL was positively associated with APACHE II score, SOFA score, CRP, PCT, TNF-α, and IL-1β levels, and mortality rates.	(61)
XIST	↓	male SD rats	HSAECs, HEK-293T	miR-16-5p	_	Up-regulation of XIST increased viability and inhibited inflammatory response and apoptosis in sepsis-induced ALI.	(62)
XIST	↓	CLP-induced AKI mice	HK-2, TCMK-1	↑ miR-155-5p, ↓ WWC1	_	Up-regulation of XIST decreased sepsis-induced AKI.	(63)
XIST	↑	30 patients and 10 HCs, male SD rats	Kupffer	↑ BRD4	_	Downregulation of XIST reduced inflammation, oxidative stress, and apoptosis in sepsis-induced acute liver injury.	(64)
XIST	↑	GEO database: GSE94717 ( 6 patients with sepsis-induced AKI and 6 HCs)	MPC5	↓ miR-15a-5p, ↑ CUL3	_	Up-regulation of XIST enhanced apoptosis in sepsis-induced AKI.	(65)
xist	↑	_	MCM	↓ PGC-1α, ↓ Tfam	_	Downregulation of xist inhibited apoptosis and induced proliferation.	(66)
GAS5	↓	60 patients with sepsis and 60 HCs	AC16	↓ miR-214	_	Downregulation of GAS5 restrained apoptosis of cardiomyocytes induced by LPS. GAS5 could regulate miR-214 through methylation pathway.	(67)
CRNDE	↓	male specific-pathogen-free Wistar rats	_	↑ miR-29a, ↓ SIRT1	↑ NF-κB/PARP1 signaling	Up-regulation of CRNDE reduced apoptosis, oxidative stress and inflammatory response.	(68)
CRNDE	↑	136 patients with sepsis and 151 HCs	THP-1	↓ miR-181a-5p, ↑ TLR4	_	Up-regulation of CRNDE was correlated with poorer OS and was a significant predictor in patients with sepsis. Downregulation of CRNDE reduced sepsis-related inflammatory pathogenesis.	(69)
CRNDE	↑	male C57 mice	_	↑ p65	↑ TLR3/NF-κB pathway	Downregulation of CRNDE reduced edema, necrosis and apoptosis in sepsis-induced AKI.	(70)
CRNDE	↑	_	HK-2	↓ miR-146a	↑ TLR4/NF-κB signaling pathway	Up-regulation of CRNDE enhanced cell injuries, inflammatory responses and apoptosis in sepsis-induced AKI.	(71)
CRNDE	↓	rats	HK-2, HEK293	↑ miR-181a-5p, ↓ PPARα	_	Downregulation of CRNDE increased the urea nitrogen and serum creatinine, and reduced proliferation and promoted apoptosis.	(72)
CRNDE	↓	male SD rats	L02	↑ miR-126-5p, ↓ BCL2L2	_	Up-regulation of CRNDE increased viability and repressed apoptosis in sepsis-induced liver injury.	(73)
HOTAIR	↓	male SD rats	HK-2	↑ miR-34a, ↓ Bcl-2	_	Up-regulation of HOTAIR reduced apoptosis in sepsis-induced AKI.	(74)
HULC	↑	110 patients with sepsis and 100 HCs	HMEC-1, CRL-3243	↓ miR-128-3p, ↑ RAC1	_	Downregulation of HULC restrained apoptosis and inflammation, and protected HMEC-1 cells from LPS-induced injury.	(75)
HULC	↑	174 patients with sepsis and 100 HCs	_	_	_	Expression of HULC was correlated with APACHE II, SOFA score, and 28‐day deaths. It was also positively associated with Scr, WBC, and CRP, but negatively correlated with albumin.	(76)
HULC	↑	56 patients with sepsis and 56 HCs	HUVECs	↓ miR-204-5p, ↑ TRPM7	_	Downregulation of HULC promoted viability and reduced apoptosis, inflammatory responses and oxidative stress.	(77)
HULC	↑	C57BL/6 mice	HMECs	↑ IL6, ↑ ICAM1, ↑ VCAM1	_	Downregulation of HULC reduced levels of pro-inflammatory factors.	(78)
TapSAKI	↑	SD rats	HK-2	↓ miR-22	↑ TLR4/NF-κB pathway	Downregulation of TapSAKI decreased inflammatory factors and renal function indicators, so decreased kidney injury.	(79)
ITSN1‐2	↑	309 patients with intensive care unit (ICU)‐treated sepsis and 300 HCs	_	_	_	High levels of ITSN1‐2 were correlated with elevated disease severity, inflammation, and poor prognosis in sepsis patients.	(80)
LincRNA-p21	↑	sepsis-induced ALI rat model	BEAS-2B c	_	_	Downregulation of LincRNA-p21 restrained apoptosis, inflammatory responses and oxidative stress in sepsis-induced ALI.	(81)
TCONS_ 00016233	↑	15 patients with septic AKI and non-AKI, and 15 HCs, C57BL/6J mice	HK-2	miR-22-3p, ↑ AIFM1	TLR4/p38MAPK axis.	Downregulation of TCONS_00016233 restrained LPS-induced apoptosis. Up-regulation of TCONS_00016233 induced LPS-induced apoptosis and inflammatory responses.	(82)
UCA1	↑	C57BL/6 mice	HMECs	↑ IL6, ↑ ICAM1, ↑ VCAM1	_	Downregulation of UCA1 reduced inflammatory responses.	(78)
NR024118	↓	82 patients with sepsis without MD, 35 patients with sepsis and MD and 82 HCs	AC16	↑ IL-6	NF-κB signaling pathway	Up-regulation of NR024118 reduced the secretion of IL-6 and apoptosis, and improved LPS-induced myocardial APD duration and cell injury.	(83)
MIR155HG	↑	28 patients with sepsis and 28 without sepsis	HL-1, RAW 264.7	↓ miR-194-5p, ↑ MEF2A	_	Downregulation of MIR155HG increased viability and decreased apoptosis and inflammatory responses.	(84)
LUCAT1	↑	GEO dataset: GSE101639	H9C2	↓ miR-642a, ↑ ROCK1	_	Downregulation of LUCAT1 decreased inflammatory responses.	(85)
SOX2OT	↑	male C57B6/L mice	H9c2	↑ SOX2	_	Downregulation of SOX2OT reduced mitochondrial dysfunction in septic cardiomyopathy. Overexpression of SOX2OT aggravated mitochondrial dysfunction in septic cardiomyopathy	(86)
MEG3	↑	male C57BL/6 mice	TECs	↓ miR-18a-3P	_	Downregulation of MEG3 reduced number of pyroptotic cells, secretion of LDH, IL-1β, and IL-18, and expression of GSDMD in LPS-induced AKI.	(87)
MEG3	↑	82 patients with sepsis and 54 HCs	Human primary renal mixed epithelial cells , AC16	_	_	Patients with high levels of MEG3 showed higher mortality rate, and downregulation of it inhibited apoptosis induced by LPS.	(88)
MEG3	↑	112 patients with sepsis and 100 HCs	_	_	_	High levels of MEG3 were associated with 28‐day deaths and it was found to be a predictor of higher ARDS risk.	(89)
MEG3	↑	219 patients with sepsis and 219 HCs, male C57BL/6 J mice	_	↓ miR‐21	_	Lnc‐MEG3 expression was positively correlated with cardiomyopathy, APACHE II score, SOFA score, Scr, TNF‐α, IL‐1β, IL‐6, and IL‐17, 28‐day deaths, while negatively correlated with albumin.	(90)
MEG3	↓	male C57/BL mice	Caco2	↑ miR-129-5p, ↓ SP-D	_	Overexpression of MEG3 reduced villus length and apoptosis, inhibited intestinal injury and enhanced proliferation.	(91)
GAS5	↓	_	conditional immortalized podocyte line	↓ PTEN	↑ PI3K/AKT pathway	Downregulation of GAS5 elevated the Podocyte Injury.	(92)
LINC00472	↑	male SD rats	THLE-3	↓ miR-373-3p, ↑ TRIM8	_	Downregulation of LINC00472 enhanced viability and suppressed apoptosis.	(93)
HOTAIR	↑	male e C57B6/L mice	HL-1	↑ p-p65, ↑ NF-κB	NF-κB pathway	Downregulation of HOTAIR restrained LPS-induced myocardial dysfunction in septic mic. HOTAIR was involved in p65 phosphorylation and NF-κB activation, leading to 15 TNF-α production.	(94)
HOTAIR	↑	male SD rats	HK-2	↓ miR-22, ↑ HMGB1	_	Downregulation of HOTAIR reduced renal function indicators (blood urea nitrogen and serum creatinine).	(95)
Hotairm1	↑	male C57BL/6 mice	MDSCs	↑ S100A9 localization	_	Downregulation of Hotairm1 restrained the suppressive functions of late sepsis Gr1+CD11b+ MDSCs. Hotairm1 Was involved in shuttling S100A9 protein to the nucleus.	(96)
NKILA	↑	_	HK2	↓ miR-140-5p, ↑ CLDN2	_	Downregulation of NKILA restrained apoptosis, autophagy and inflammation and promoted viability in sepsis-induced AKI.	(97)
HOXA‐AS2	↓	44 patients with sepsis and 44 HCs, adults clean Kunming mice	HK‐2	↑ miR‐106b‐5p	↑ Wnt/β‐catenin and NF‐κB pathways	Up-regulation of HOXA‐AS2 increased viability and repressed apoptosis and protect cells to resist LPS‐induced damage in sepsis-induced AKI.	(98)
SNHG14	↑	_	HK-2	miR-93, ↑IL-6R, ↑IRAK4	TLR4/NF-κB pathway, ↑ NF-κB and STAT3 signaling	Up-regulation of SNHG14 promoted oxidative stress, inflammation, and apoptosis. TLR4/NF-κB pathway induced upregulation of SNHG14.	(99)
lncRNA-CCL2	↑	male C57BL/6 mice	_	↓ SIRT1	_	Expression of lncRNA-CCL2 was inhibited by SIRT1 through maintaining a more repressive chromatin state in lncRNA-CCL2 locus. Downregulation of SIRT1 induced inflammatory response.	(100)
DLX6-AS1	↑	patients with septic AKI	HK-2	↓ miR-223-3p, ↑ NLRP3	_	Downregulation of DLX6-AS1 suppressed LPS-induced cytotoxicity and pyroptosis. Expression of DLX6-AS1 was positively correlated with levels of creatinine in the serum of patients.	(101)
CASC2	↓	_ patients with sepsis and HCs	HK-2	↑ miR-155	↑ NF-κB signaling pathway	The levels of CASC2 were negatively correlated with the severity of AKI. CASC2 expression induced cell viability and inhibited inflammatory response, apoptosis and oxidative stress.	(102)
CASC2	↓	patients with sepsis and HCs	HPAEpiC	↑ miR-152-3p, ↓ PDK4	_	Up-regulation of CASC2 increased viability and restrained apoptosis, inflammatory and oxidative damages.	(103)
ZFAS1	↓	202 patients with sepsis and 200 HCs	_	_	_	Expression of ZFAS1 was negatively associated with APACHE II, level of CRP, TNF-α, IL-6 and positively with IL-10.	(104)
ZFAS1	↓	male SD rats	H9C2	↑ miR-34b-5p, ↓ SIRT1	_	Up-regulation of ZFAS1 decreased inflammatory responses and apoptosis.	(105)
ZFAS1	↑	male C57BL/6 mice	_	↓ miR-590-3p, SP1	AMPK/mTOR signaling	Downregulation of ZFAS1 reduced LPS-induced pyroptosis and enhanced LPS-suppressed autophagy in sepsis-induced cardiac dysfunction.	(106)
ZFAS1	↓	22 patients with SIMI and 24 HCs, rats treated by LPS	H9C2	↑ miR-138–5p, ↓ SESN2	_	Up-regulation of ZFAS1 attenuated myocardial injury and inflammatory response.	(107)
Mirt2	↓	male SD rats	_	↑ MiR-101	↓ PI3K/AKT Signaling Pathway	Up-regulation of Mirt2 inhibited inflammatory responses and improved cardiac function.	(108)
Mirt2	↓	40 patients with sepsis, 40 patients with sepsis‐ALI, 40 HCs	HBEpCs	↓ miR‐1246	_	Up-regulation of Mirt2 inhibited LPS‐induced inflammatory response, apoptosis, and promoted miR‐1246 expression but reduced its gene methylation.	(109)
TCONS_00016406	↓	male C57BL/6 mice	PTEC	↑ miR-687, ↓ PTEN	_	Up-regulation of lncRNA 6406 inhibited inflammatory responses, apoptosis and oxidative stress in LPS-induced AKI.	(110)
NORAD	↑ in NS patients	88 patients with late-onset NS and 86 patients with pneumonia neonates	RAW264.7	↓ miR-410-3p	_	Expression of NORAD was closely correlated with WBC, PCT, IL-6, IL-8, and TNF-α.	(111)
GAS5	↑	_	THP-1	↓ miR-23a-3p, ↑ TLR4	_	Downregulation of GAS5 inhibited inflammation and apoptosis.	(112)
lnc‐ANRIL	↑	126 patients with sepsis and 125 HCs	_	↓ miR‐125a	_	lnc‐ANRIL showed good predictive values for sepsis risk. lnc‐ANRIL was positively associated with CRP and PCT levels, disease severity scale scores, and pro‐inflammatory cytokine levels, 28‐day deaths in sepsis patients,	(113)
PVT1	↑	109 patients with sepsis and 100 HCs	_	_	_	PVT1 was found to be an independent risk factor for sepsis ARDS. And PVT1 expression positively associated with disease severity and 28-day deaths.	(114)
PVT1	↑	_	THP-1	_	↑ p38 MAPK signaling pathway	Downregulation of PVT1 reduced levels of IL-1β and TNF-α mRNA and inhibited the p38 MAPK signaling pathway,	(115)
PVT1	↑	sepsis model mice	HK-2	↓ miR-20a-5p, ↑ NLRP3	_	Downregulation of PVT1 inhibited pyroptosis in septic AKI.	(116)
PVT1	↑	Mice model with sepsis	_	↓ miR-29a, ↑ HMGB1	_	Downregulation of PVT1 reduced LPS-induced myocardial injury and alleviated M1 macrophage polarization.	(117)
HOTAIR	↑	C57BL/6 mice	Monocytes	↓ miR-211	_	Overexpression of HOTAIR suppressed proliferation and promoted apoptosis.	(118)
HOTAIR	↑	LPS-induced septic cardiomyopathy mice	H9C2	↑ PDCD4, Lin28	_	Downregulation of HOTAIR inhibited inflammatory responses and apoptosis.	(119)
DILC	↓	18 patients with sepsis and 18 HCs	PBMCs, THP-1	↑ IL-6	_	DILC suppressed the transcription of IL-6, DILC decreased levels of STAT3, p-STAT3, TLR4, TNF-α, CCL5, E-selectin and CXCR1.	(120)
RMRP	↑	C57BL/6 mice	HK-2	↓ miR-206, ↑ DDX5	_	Downregulation of RMRP inhibited inflammatory response and apoptosis in sepsis-induced AKI.	(121)
GAS5	↑	C57BL/6 mice	_	↓ miR-449b, ↑ HMGB1	↑ HMGB1/NF-κB pathway	Downregulation of GAS5 inhibited pro-inflammatory reaction and alleviated myocardial injury.	(122)
TapSAKI	↑	_	HK-2	↓ miR-205, ↑ IRF3	_	Downregulation of TapSAKI alleviated LPS-induced damage.	(123)
SNHG16	↑	male SD rats	BEAS-2B	↓ miR-128-3p, ↑ HMGB3	_	Downregulation of SNHG16 reduced the apoptosis and inflammation in sepsis-induced ALI.	(124)
DANCR	↓	20 patients with sepsis-induced AKI and 20 HCs	HK-2	↑ miR-214, ↑ KLF6	_	Up-regulation of DANCR promoted viability and suppressed cell apoptosis and inflammatory responses.	(125)
CASC2	↓	_	HK2, HEK293	↑ miR-545-3p to regulate, ↓ PPARA	_	Up-regulation of CASC2 increased viability and inhibited apoptosis, migration, epithelial-mesenchymal transition and oxidative stress.	(126)
SNHG1	↓	_	H9c2	↑ miR-181a-5p, ↓ XIAP	_	Up-regulation of SNHG1 increased viability and inhibited inflammatory responses and oxidative stress.	(127)
SNHG14	↑	_patients with sepsis	HK-2	↓ miR-495-3p, ↑ HIPK1	_	SNHG14 is upregulated in patients. SNHG14 prevented proliferation and autophagy and boosted apoptosis and inflammatory responses.	(128)
Linc-KIAA1737–2	↑	_	HK-2	↓ MiR-27a-3p	_	Downregulation of Linc-KIAA1737–2 reduced apoptosis.	(129)
PlncRNA-1	↓	6 patients with septic AKI and 6 HCs	NRK-52E	↓ BCL2	_	Up-regulation of PlncRNA-1 meliorated proliferation and prevented apoptosis and autophagy.	(130)
CDKN2B-AS1	↑	sepsis patients 47 and 55 HCs	BEAS-2B	↓ miR-140-5p , ↑ TGFBR2	↑ TGFBR2/smad3 pathway	Downregulation of CDKN2B-AS1 promoted viability reduced apoptosis and inflammation.	(131)

ARDS, acute respiratory distress syndrome; HCs, healthy controls; ALI, acute lung injury; LPS, lipopolysaccharide; SD, Sprague–Dawley; AKI, acute kidney injury; SOFA, sequential organ failure assessment; Scr, serum creatinine; WBC, white blood cell; CRP, C-reactive protein; PBMCs, peripheral blood mononuclear cells; PCT, procalcitonin; APACHE, physiology and chronic health evaluation; MPO, Myeloperoxidase; NS, Neonatal sepsis; SIMI, sepsis-induced myocardial injury.

## miRNAs and Sepsis

miRNAs have sizes about 22 nucleotides and regulate expression of genes through binding with different regions of target mRNAs, particularly their 3’ UTR. They can either degrade target mRNA or suppress its translation. Several miRNAs have been found to influence course of sepsis. Altered expression of these small-sized transcripts has been reported in sepsis by numerous research groups. For instance, plasma levels of miR-494-3p have been shown to be decreased in sepsis patients compared with healthy controls in correlation with up-regulation of TLR6. Expression level of miR-494-3p has been decreased in LPS-induced RAW264.7 cells, parallel with up-regulation of TLR6 and TNF-α. Forced over-expression of miR-494-3p in RAW264.7 cells could reduce TNF-α level and suppress translocation of NF-κB p65 to the nucleus. TLR6 has been shown to be targeted by miR-494-3p. Taken together, miR-494-3p could attenuate sepsis-associated inflammatory responses through influencing expression of TLR6 (132). miR-218 is another miRNA which participates in the pathoetiology of sepsis. This miRNA could reduce inflammatory responses in the sepsis through decreasing expression of VOPP1 *via* JAK/STAT axis (133).

miR-122 is another important miRNA in the sepsis which has superior diagnostic power compared with CRP and total leucocytes count for distinguishing sepsis from wound infection. miR-122 has also been found to be a prognostic marker for sepsis, albeit with poor specificity and accuracy values (134).

In the mice model of sepsis, decreased levels of miR-208a-5p and increased levels of SOCS2 has been associated with enhanced activity of SOD, while reduction in LDH and MDA activities. Moreover, down-regulation of miR-208a-5p has been associated with low levels TNF-α, IL-6, NF-κB p65 and HIF-1α in this animal model. miR-208a-5p silencing could decrease the extent of mitochondria swelling, and inhibit apoptosis of cardiomyocytes in animal model of sepsis. Taken together, miR-208a-5p suppression has been suggested as a modality to attenuate sepsis-related myocardial damage. This function is mediated through NF-κB/HIF-1α axis (135).

miR-21 is another miRNA whose role in sepsis has been investigated by several groups. Down-regulation of miR-21 has been shown to inhibit inflammasome activation, ASC pyroptosome, LPS-induced pyroptosis and septic shock in one study (136). On the other hand, another study in animal models of sepsis has shown that up-regulation of miR-21 reduced inflammation and apoptosis (137). Similarly, βMSCs-derived exosomes have been shown to reduce symptoms in septic mice and improve their survival rate through up-regulation of miR-21 (138).

**Figure 3:**
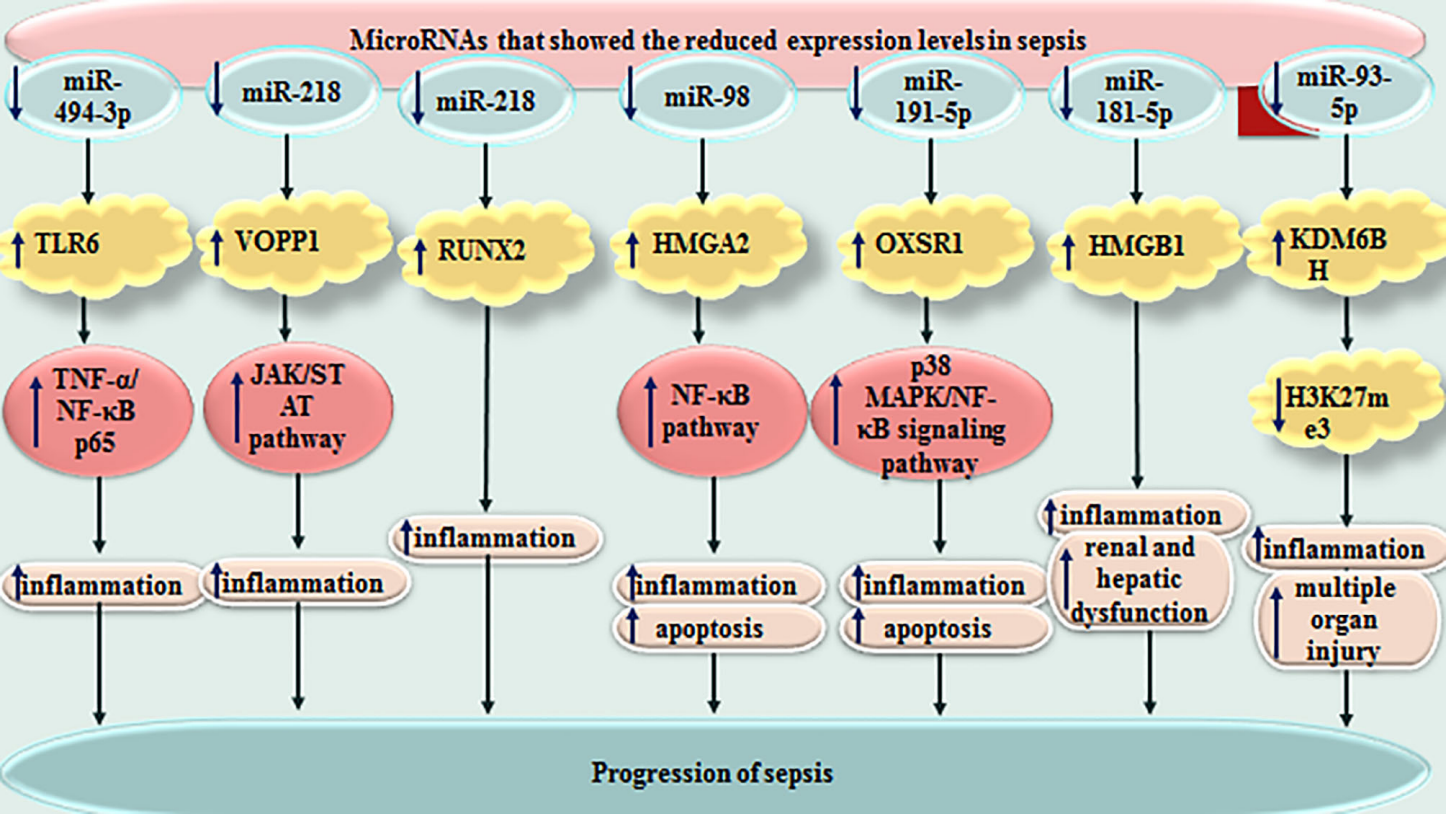
Down-regulated miRNAs in sepsis.

miR-328 is another miRNA which is dysregulated in sepsis patients as well as animal models of sepsis. Serum levels of this miRNA could properly differentiate sepsis from normal conditions. Thus, miR-328 has been suggested as a diagnostic biomarker for sepsis. Moreover, down-regulation of miR-328 could amend sepsis-related heart dysfunction and inflammatory responses in this tissue (139). miR-452 is another miRNA with diagnostic applications in sepsis. Notably, serum and urinary levels of this miRNA have been suggested as possible markers for early diagnosis of sepsis-associated acute kidney injury, since expression of this miRNA has been higher in sepsis patients with acute kidney injury compared with those without this condition (140) (**Table 2**). **Figure 3** depicts miRNAs that are down-regulated in sepsis.

**Table 2:** Lists the function of miRNAs in the course of sepsis.

miRNA	Pattern of Expression	Clinical Samples/Animal Model	Assessed Cell Lines	Targets / Regulators	Signaling Pathways	Description	Reference
miR-15a-5p	↑	GEO database: GSE94717 (6 patients with sepsis-induced AKI and 6 HCs)	MPC5	↓ XIST, ↓ CUL3	_	Downregulation of miR-15a-5p reduced apoptosis in sepsis-induced AKI.	(65)
miR-494-3p	↓	_Patients with sepsis and HCs	RAW264.7	↑ TLR6	_	Upregulation of microRNA-494-3p reduced inflammation, TNF-α level, and prevented nuclear translocation of NF-κB p65.	(132)
miR-218	↓	53 Patients with sepsis and 20 HCs, septic mouse model	PBMCs	↑ VOPP1	↑ JAK/STAT pathway	Upregulation of microRNA-494-3p reduced inflammation.	(133)
miR-218	↓	male S SD rats	RAW264.7	↑ RUNX2		Up-regulation of miR-218 inhibited inflammatory response.	(141)
miR-122	↑	25 patients with sepsis and 25 patients with local wound infections as a control group	_	_	_	miR-122 showed higher AUC in comparison with CRP and TLC which had 66.6% sensitivity, 50% specificity, and 56.0% accuracy as a prognostic biomarker for sepsis.	(134)
miR-208a-5p	↑	septic mouse model	_	↓ SOCS2	↑NF-κB/HIF-1α pathway	Downregulation of miR-208a-5p decreased reduced degree of mitochondria swelling, and inhibited apoptosis.	(135)
miR-328	↑	110 Patients with sepsis and 89 HCs, male SD rats	_	_	_	miR-328 expression was positively associated with Scr, WBC, CRP, PTC, APACHE II score, and SOFA score. miR-328 was found to be a good diagnostic value for sepsis. Downregulation of miR-328 reduced inflammatory response.	(139)
miR-452	↑	47 sepsis patients with AKI, 50 patients without AKI, and 10 HCs	BUMPT	NF-KB	_	Serum and urinary miR-452 could be a potential biomarker for early detection of septic AKI. It was upregulated in sepsis patients with AKI compared with without AKI. miR-452 had high diagnostic value for AKI.	(140)
miR‐21	↓	219 Patients with sepsis and 219 HCs	_	_	_	miR‐21 was found to be a good value in predicting sepsis risk. miR‐21 expression was negatively correlated with APACHE II, SOFA score, and 28‐day mortality risk.	(142)
miR‐126	↑	208 Patients with sepsis and 210 HCs	_	_	_	miR‐126 expression was positively correlated with APACHE II, serum creatinine, CRP, TNF‐α, IL‐6, IL‐8, mortality rate, but negatively with IL‐10.	(143)
mir-103	↓	196 Patients with sepsis and 196 HCs	_	_	_	mir-103 predicted high ARDS risk. Mir-103 and was negatively associated with APACHE II score, SOFA score, serum creatinine, CRP, TNF, IL- 1β, IL-6, IL-8, 28-day deaths, but positively correlated with albumin.	(144)
mir-107	↓	196 Patients with sepsis and 196 HCs	_	_	_	mir-107 predicted high ARDS risk. mir-107 and was negatively associated with APACHE II score, SOFA score, serum creatinine, CRP, TNF, IL- 1β, IL-6, IL-8, 28-day deaths, but positively correlated with albumin	
miR-92a	↑ in sepsis-induced ARDS	53 sepsis patients (36 patients with sepsis-induced ARDS)	HPMEC, A549	_	↓ Akt/mTOR signaling pathway	Downregulation of mir-92a reduced apoptosis and inflammatory response, and enhanced migration	(145)
miR-98	↓	male C57BL/6 mice	_	↑ HMGA2	↑ NF-κB pathway	Upregulation of miR-98 prevented HMGA2, NF-κB, TNF-α, IL-6, Bcl-2 and augmented IL-10, Cleaved caspase-3 and Bax expression, it reduced LVEDP, CTn-I, BNP, ALT, AST, TBIL, LDH, and PaCO2 but elevated +dp/dt max, -dp/dt max, pH and PaO2.	(146)
miR‐125a	↑	150 Patients with sepsis and 150 HCs	_	_	_	miR‐125a expression was positively associated with Scr, APACHE II score, SOFA score.	(147)
miR‐125b	↑	150 Patients with sepsis and 150 HCs	_	_	_	miR‐125b was correlated with Scr, CRP, APACHE II score, SOFA score, and chronic obstructive pulmonary disease , and 28-day deaths.	
miR-199a	↑	male C57BL/6 mice	_	↓ SIRT1	_	Downregulation of miR-199a reduced apoptosis and inflammatory response.	(148)
miR-495	↓	105 Patients with sepsis and 100 HCs, rats	_	_	_	miR-495 was negatively correlated with Scr, WBC, CRP, PCT, APACHE II score and SOFA score. CLP rats showed worse LVSP, LVEDP, ± dp/dtmax, and exhibited an increase in serum CTn-I, CK-MB, TNF-α, IL-6 and IL-1β.	(149)
miR-106a	↑	50 patients with sepsis and 30 HCs, clean Kunming mice	TCMK-1	↓ THBS2	_	Downregulation of miR-106a reduced apoptosis and inflammatory response.	(150)
miR‐146a	_	male C57BL/6 mice	MSCs	IL‐1β	–	IL‐1β stimulation resulted in packaging miR‐146a into exosomes. The exosomal miR‐146a was transferred to macrophages, yielded to M2 polarization, and finally led to high survival in septic mice.	(151)
miR-574	↓	CLP-treated mice	HBE	↑ C3	_	Upregulation of mir-574 increased viability, inhibited apoptosis, and reduced sepsis-induced ERS.	(152)
miR-195	_	wistar rats with sepsis		_	TGF-β1/Smads signaling pathway,	MicroRNA-195 could promote cardiac remodeling by up-regulating the nanoantibiotics signaling pathway in sepsis rats.	(153)
miR-133a	↑	septic mouse model	RAW264.7	↓ SIRT1	_	Downregulation of miR-133a prevented inflammatory response, sepsis-induced lung, liver and kidney injuries.	(154)
miR-191-5p	↓	female Wistar rats	_	↑ OXSR1	↑ p38 MAPK/NF-κB signaling pathway	Upregulation of miR-191-5p prevented inflammatory response and apoptosis in	(155)
miR-146a	↑	180 patients with sepsis and 180 HCs	_	_	_	MiR-146a was of good value in predicting high sepsis risk and 28-day mortality risk. MiR-146a was positively associated with biochemical indices, inflammatory cytokines, overall disease severity.	(156)
miR-146b	↑	180 patients with sepsis and 180 HCs	_	_	_	miR-146b was of good value in predicting high sepsis risk and 28-day mortality risk. MiR-146a was positively associated with biochemical indices, inflammatory cytokines, and overall disease severity. .	
miR-126	↓	20 patients with sepsis and 30 patients with general infection	_	_	_	miR-126 was negatively associated with the levels of caspase-3, APACHE II score, and positively with 28-day cumulative survival rate. AUC for predicting the prognosis by miR-126 was 0.823.	(157)
miR-223	_	C57BL/6 mice	RAW264.7	_	_	Upregulation of mir-223 impelled M2 macrophage through lower activity of glycolysis Pathway. the Implementation of miR-223 over-expressed macrophages with IL-4 pre-conditioning alleviated sepsis severity.	(158)
miR-146b	↓	septic mouse model	HK-2	↑ IRAK1	↑ NF-κB pathway	Treatment with hucMSC-Ex improved survival in mice with sepsis by reducing levels of IRAK1, increasing of miR-146b level, and inhibition of NF-κB activity.	(159)
miR-1-3p	↑	male SD rats	HUVECs	↓ SERP1	_	miR-1-3p decreased proliferation, and increased apoptosis, and permeability and HUVECs membrane injury.	(160)
miR-25	↓	70 patients with sepsis and 30 patients with SIRS	_	_	_	Levels of miR-25 was negatively associated with the severity of sepsis, SOFA score, CRP and PCT level, 28-day deaths, and levels of oxidative stress indicators.	(161)
miR-370-3p	↑ in SAE	12 patients with sepsis without encephalopathy, 17 patients with SAE, 20 patients with severe uremia and 12 HCs , male C57BL/6 mice	_	_	_	miR-370-3p was associated with TNF-α and increased brain apoptosis in SAE mice.	(162)
miR-21	↑	GEO database: GSE26440 (88 children with septic shock and 26 HCs), C57BL/6 mice	_	↓ A20, ↑ NLRP3	↑ NF-κB pathway	Downregulation of miR-21 inhibited inflammasome activation, ASC pyroptosome, LPS-induced pyroptosis and septic shock.	(136)
miR-21	↓	CLP mouse model	_	↑ PDCD4, ↑ PTEN	PDCD4/NF-κB and PTEN/AKT pathways	rIPC protected kidneys from injury by miR-21. miR-21 was transported from ischemic limbs to the kidneys by exosomes.	(163)
miR-21	↓	septic mouse model	MTEC	↑ PDCD4	↑ NF-κB pathway	Upregulation of miR-21 reduced inflammation and apoptosis.	(137)
miR-21	_	septic mice	_	_	_	Hyperoside decreased miR-21 levels so reduced inflammatory responses and increased viability.	(164)
miR-21	↓	_	MSCs	↑ PDCD4	_	βMSCs-derived exosomes reduced symptoms in septic mice and improved their survival rate through miR-21 upregulation.	(138)
miR-21	↑	septic C57BL/6J mice	_	↓ PGE2, ↓ IL-10	_	Downregulation of miR-21 reduced bacterial growth, systemic inflammation, organ damage, macrophage glycolysis, and increased animal survival.	(165)
miR-21-3p	↑	SD rats	TECs	↓ AKT, ↓ CDK2, ↑ FOXO1	–	miR-21-3p regulated lipid metabolism and increased cell cycle arrest and apoptosis.	(166)
miR-34	↑	male C57BL/6 mice (15 control group and 15 sepsis model group)	_	↓ KLF4	_	Plasma miR-34a was positively associated with SCr and BUN.	(167)
miR-483-5p	↑	CLP-treated mice	PMVECs	↓ PIAS1	_	Downregulation of miR-483-5p reduced inflammation and apoptosis and improved lung injury in mice with sepsis-induced ALI.	(168)
miR-181-5p	↓	CLP- treated mice	_	↑ HMGB1	_	Upregulation of miR-181-5p reduced inflammatory response, and sepsis-induced renal and hepatic dysfunction.	(169)
miR-20a	_	SD rats	_	_	_	miR-20a could deteriorated AKI via activating autophagy in sepsis rats.	(170)
hsa-miR-92a-3p	↓ in sepsis-induced coagulopathy group	116 patients with sepsis	_	_	_	AUC of hsa-mir-92a-3p was 0.660. Levels of plasma hsa-mir-92a-3p were related to plasma lipocalin-2 level, activated partial thromboplastin time, and prothrombin activity.	(171)
miR-93-5p	↓	septic mouse model	HK2	↑ KDM6B, ↓ H3K27me3	_	Extracellular vesicles containing miR-93-5p reduced inflammation, apoptosis, multiple organ injury, and vascular leakage in septic mice.	(172)
miR-223	↓	143 patients with sepsis and 44 HCs	_	_	_	Expression of miR-223 was negatively correlated with SOFA scores and positively with survival rate. Upregulation of miR-223 decreased apoptosis and increased proliferation and G1/S transition.	(173)
miR-34a	↑	male C57BL/6 mice	_	↓ SIRT1, ↓ ATG4B	_	Downregulation of miR-34a reduced inflammatory response and pyroptosis, apoptosis and enhanced autophagy.	(174)
miR-30a	↑	septic rats	_	↓ SOCS-1	↑ JAK/STAT signaling pathway	Upregulation of miR-30a promoted apoptosis and inhibited proliferation.	(175)
miR-150-5p	↓	rat septic shock model	H9C2	↑ Akt2	_	Upregulation of miR-150-5p inhibited apoptosis.	(176)
miR-140	↓	SPF male BALB/c mice	_	_	↑ WNT signaling pathway	Upregulation of miR-140 inhibited apoptosis and inflammation, skeletal muscle glycolysis and atrophy.	(177)
miR-22-3p	↓	male SD rats	HK-2	↑ HMGB1, ↑ PTEN	_	Upregulation of miR-22-3p inhibited apoptosis and inflammatory response	(178)
miR-205-5b	↑	BALB/c mice	RAW264.7	HMGB1	_	Down regulation of miR-205-5b increased HMGB1 expression in LPS-induced sepsis.	(179)
miR-526b	↓	BALB/c mice	HK2	↑ ATG7	_	Upregulation of miR-526b increased viability by inhibiting autophagy.	(180)
miR-145a	↓	septic mouse model	_	↑ Fli-1	↑ NF-κB signaling	Upregulation of miR-526b reduced levels of proinflammatory cytokines.	(181)
miR‐125a	↑	150 patients with sepsis and 150 HCs	_	_	_	AUC of miR‐125a: 0.749 miR‐125a was positively correlated with APACHE II score and SOFA score.	(182)
miR‐125b	↑	150 patients with sepsis and 150 HCs	_	_	_	AUC of miR‐125b: 0.839 miR‐125b was positively correlated with APACHE II score, SOFA score CRP, TNF‐α, IL‐6, IL‐17, IL‐23, and 28‐day mortality risk.	
miR-122	↑	108 patients with sepsis and 20 patients with infections without sepsis as controls	_	_	_	AUC of miR-122: 0.760 miR-122 was found as independent prognostic factor for 30-day mortality.	(183)
miR-135a	↑	_patients with sepsis and HCs, BALB/c mice	_	_	↑ p38 MAPK/NF-κB pathway	Upregulation of miR-135a exacerbated inflammation and myocardial dysfunction.	(184)
miR-133a	↓	_	TCMK-1	↑ BNIP3L	↑ NF-κB pathway	Upregulation of miR-133a reduced inflammation and apoptosis.	(185)
miR-223	_	male C57BL/6 mice	_	_	_	In multiple models of experimental sepsis, miR-223 showed the complex role in the pathogenesis of septic kidney injury.	(186)
miR-155	↑	44 patients with severe sepsis, 102 patients with sepsis, and 19 HCs	↑	↑	↑	AUC of miR-155: 0.782 (for predicting 30-day mortality in ALI)	(187)
miR-146a	↑	44 patients with severe sepsis, 102 patients with sepsis, and 19 HCs	↑	↑	↑	AUC of miR-146a: 0.733 (for predicting 30-day mortality in ALI), CC genotype of rs2910164 in miR-146a was correlated with worse treatment result.	
miR-194	↑	_	H9c2	↓ Slc7a5	↑ Wnt/β-catenin pathway	Upregulation of miR-194 increased apoptosis.	(188)
miR-30a	↑	male C57BL/6 mice	RAW 264.7	↓ ADAR1, ↓ SOCS3	_	Upregulation of ADAR1 (a target of miR-30a) reduced inflammation and organ damage.	(189)
miR-27b	↓	male C57BL/6 mice	BMMSCs	↑ JMJD3	↑ NF-κB signaling pathway	Upregulation of miR-27b MSC-derived exosomes reduced pro-inflammatory cytokines.	(190)
miR-155	↑	BALB/c mice	_	↓ SOCS1	↑ JAK/STAT signaling	Downregulation of miR-155 alleviated LPS-induced mortality and liver injury	(191)
miR-155	↓	C57BL/6 mice	_	↑ Arrb2	↑ JNK signaling pathway	Upregulation of miR-155 ameliorated late sepsis survival and its cardiac dysfunction, and reduced pro-inflammatory responses.	(192)
miR-155	↑	_patients with sepsis and HCs, mouse septic shock model	_	↓ CD47	_	Downregulation of microRNA-155 reduced sepsis-associated cardiovascular dysfunction and mortality.	(193)
miR-155	↑	60 patients with sepsis and 20 HCs	_	↑ Foxp3	_	Expression of miR-155 was correlated with APACHEII score, it was significantly higher in non-survival group.	(194)
miR-155	↑ in sepsis and ALI/ARDS than sepsis but no ALI/ARDS	156 patients with sepsis (41 with ALI and 32 with ARDS)	_	_	_	AUC of miR-155: 0.87, miR-155 was positively associated with IL-1β, TNF-α levels, and ALI/ARDS score, but negatively with PaO2/FiO2.	(195)
miR-29c-3p	↑	86 patients with sepsis and 85 HCs, male SD rats	_	_	_	AUC of miR-29c-3p: 0.872 miR-29c-3p expression was positively correlated with APACHE II score, SOFA score, levels of CRP and PCT. miR-29c-3p was found to be an independent factor in the occurrence of cardiac dysfunction.	(196)
miR-125b	↓	40 patients with sepsis and HCs, female and male C57BL/6 mice	_	↓ PTEN, ↑ MyD88	_	PTEN increased miR125 production through associating with the nuclear localization of Drosha-Dgcr8. Downregulation of PTEN resulted in cytokine production, MyD88 abundance and mortality.	(197)
miR-203b	↓	40 patients with sepsis and HCs, female and male C57BL/6 mice	_	↓ PTEN, ↑ MyD88	_	PTEN increased miR203b production through associating with the nuclear localization of Drosha-Dgcr8. Downregulation of PTEN resulted in cytokine production, MyD88 abundance and mortality.	
miR-146	↓	_	EA. hy926	_	↑ NF-κB signaling pathway	Upregulation of reduced levels inflammatory cytokines.	(198)
miR-140-5p	↓	male SPF rats	MLE-12	↑ TLR4, ↑ MyD88	↑ NF-κB signaling pathway	Shikonin could alleviated sepsis- induced ALI by increasing the levels of miRA-140-5p and decreasing the levels of TLR4.	(199)
miR-125b	↓	male C57BL/6 mice	HUVECs	↑ ICAM-1, ↑ VCAM-1, ↑ TRAF6	↑ NF-κB signaling pathway	Upregulation of miR-125b alleviated sepsis-induced cardiac dysfunction and ameliorated survival.	(200)
miR-494	↑	ARDS rat models	_	_	↓ Nrf2 signaling pathway	Upregulation of miR-494 increased inflammatory response, oxidative stress and ALI.	(201)
miR-146a	↓	male C57BL/6 mice	H9C2, J774	↑ IRAK, ↑ TRAF6	↑ NF-κB signaling pathway	Upregulation of miR-146 reduced levels of inflammatory cytokines and sepsis-induced cardiac dysfunction	(202)
miR-223	_	221 patients with sepsis and 75 HCs, male C57Bl/6 mice	_	_	_	Levels of serum miR-223 did not differ between critically ill patients and HCs, but ICU patients with APACHE-II score had moderately decreased circulating miR-223.	(203)
miR-300	↓	septic mouse model	_	↑ NAMPT	↓ AMPK/mTOR signaling pathway	Upregulation of miR-300 increased autophagy, cell cycle entry and reduced apoptosis and inflammatory response.	(204)
miR-126	↓	male C57BL/6 mice	_	↓ HSPA12B	_	Upregulation of HSPA12B increased levels of miR-126, upregulation of miR-126 reduced levels of dhesion molecules and improved sepsis–induced cardiac dysfunction.	(205)
miR-10a	↓	62 patients with sepsis and 20 HCs	_	↑ MAP3K7	↑ NF-κB pathway	miR-10a expression was negatively association with disease severity scores, levels of c-reactive protein, procalcitonin, and 28-day death.	(206)
miR-146a	↓	mice	_	↑ Notch1	↑ NF-κB signaling	Upregulation of miR-146a reduced inflammatory responses of macrophages and protected mice from organ damage	(207)
miR-19a	↓	CLP mice	RAW 264.7	↑ Fn14	_	Upregulation of miR-19a reduced LPS-Induced Tubular Damage, it was found to protected mice from sepsis-induced AKI.	(208)
miR-214	_	male Kunming mice	_	_	_	Upregulation of miR-214 reduced apoptosis, inflammatory response, myocardial injury, and improved cardiac function in SIMI.	(209)
miR-539-5p	↓	male C57BL/6 mice	MPVECs	↑ ROCK1	_	Upregulation of miR-539-5p reduced apoptosis, inflammatory response, sepsis-induced pulmonary injury.	(210)
miR-155	↑	60 patients with sepsis and 30 HCs	_	_	_	miR-155 was positively correlated with a higher SOFA score and a greater severity. AUC of miR-155 for 28-day survival was 0.763. miR-155 derived immunosuppression through CD39(+) Tregs.	(211)
miR-146a	↑ in sepsis group compared to shame group	male BALB/C mice	_	_	_	Up-regulation of miR-146a reduced levels of inflammatory cytokine TNF-α and mitigated inflammatory reaction and lung tissue injury in sepsis-induced ALI.	(212)
miR-7110-5p	↑	52 patients with pneumonia, 44 patients with sepsis and 21 HCs	_	_	_	The sensitivity and specificity of miR-7110-5p were 84.2 and 90.5% respectively. (sepsis vs HCs)	(213)
miR-223-3p	↑	52 patients with pneumonia, 44 patients with sepsis and 21 HCs	_	_	_	The sensitivity and specificity of miR-223-3p were 82.9 and 100% respectively. (sepsis vs HCs)	
miR-19a	↑	patients with sepsis	B cells from patients with sepsis	CD22	_	Expression of CD22 initially increased but subsequently reduced. Upregulation of miR-19a resulted in an increased BCR signaling, while overexpression of CD22 reduced the effect of miR-19a and promoted its expression.	(214)
miR-206	↑	63 patients with sepsis, 30 patients with septic shock and HCs	_	_	_	miR-206 was positively associated with SOFA sore and APACHE-II score. It was observed an activated partial thromboplastin time and notably longer prothrombin time.	(215)
miR-146a	↓	male C57BL/6 mice	RAW264.7	_	↑ NF-κB signaling	Up-regulation of miR-146a reduced apoptosis, inflammatory response, and weakened organ injury in splenic macrophages.	(216)
miR-19b-3p	↓	103 patients with sepsis and 98 HCs	HUVECs	_	_	Up-regulation of miR-19b-3p reduced inflammatory response. miR-19b-3p was found to be an independent prognostic factor for 28-day survival.	(217)
miR-129-5p	↓	CLP mice	MLE-12	↑ HMGB1	_	Up-regulation of miR-129-5p reduced apoptosis, inflammatory response, , lung wet/dry weight ratio, and myeloperoxidase activity.	(218)
miR-23b	↓	30 patients with sepsis and 30 HCs	THP-1	↑ ADAM10	_	Up-regulation of miR-23b reduced apoptosis and inflammatory response.	(219)
miR-150	↓	140 patients multiple trauma and 10 HCs	MDSCs	↑ ARG1	_	Up-regulation of miR-150 reduced IL-6, TGF-β and IL-10.	(220)
miR-375	↓	_ patients with sepsis, septic mice	MDSCs	↑ miR-21	↑ JAK2/STAT3 pathway	Up-regulation of miR-375 reduced the number of sepsis Gr1+CD11b+ MDSCs in mice.	(221)
miR-31	↑	male SD rats	CACO-2	↓ HMOX1	↑ NF-κB/HIF-1α pathway	Downregulation of miR-31 reduced intestinal barrier function, intestinal mucosal permeability, oxidative damage and inflammation level.	(222)
miR-21 and miR-181b	↑ (in early sepsis) sustained (in late sepsis)	male BALB/c mice	MDSCs	↑ NFI-A	_	Down regulation of miR-21 and miR-181b decreased, immunosuppression, reprograming myeloid cells, late-sepsis mortality, and improved bacterial clearance.	(223)
miR-150	↓ slightly	223 critically ill patients (including 138 fulfilled sepsis criteria) and 76 HCs	_	_	_	serum levels of miR-150 were associated with hepatic or renal dysfunction. Low levels were correlated with an unfavorable prognosis of patients. serum levels of miR-150 were not suitable for predicting of sepsis.	(224)
miR-10a	↑	SD rats	_	_	↑ TGF-β1/Smad pathway	Up-regulation of miR-10a increased ROS, TNF-α, IL-6, and MPO, and downregulation reduced sepsis-induced liver injury.	(225)
miR-145	↓	septic mice	HUVECs	↑ TGFBR2, ↑ SMAD2, ↑ DNMT1	_	Up-regulation of miR-145 reduced LPS-induced sepsis and improved the overall survival of septic mice.	(226)
miR-150	↓	17 patients with sepsis and 32 HCs	_	_	_	Levels of miR-150 were negatively correlated with the level of disease severity, TNF-α, IL-10, and IL-18.	(227)
miR‐103a‐3p	↑	30 patients with sepsis and 30 HCs, male C57 BL/6 mice	AML12, LO2	↓ FBXW7	_	Downregulation of miR‐103a‐3p reduced apoptosis, and inflammatory response.	(228)
miR-143	↑	103 patients with sepsis, 95 patients with SIRS and 16 HCs	_	_	_	miR-143 was positively correlated with SOFA score and APACHE II score in patients with sepsis. For distinguishing between sepsis and SIRS, miR-143 showed a sensitivity of 78.6% and specificity of 91.6%.	(229)
miR-145	↓	33 patients with sepsis and 22 HCs, septic mice	BEAS-2B	↑ TGFBR2	_	Up-regulation of miR-145 reduced inflammatory response and improved the overall survival of septic mice.	(230)
miR-150	↓	C57Blk/6J mice	HPAECs	↑ Ang2	_	Downregulation of miR-150 damaged adherens junctions reannealing after injury, which caused an irreversible increase in vascular permeability. Up-regulation of miR-150 reduced vascular injury and mortality.	(231)
miR-34b-3p	↓	CLP mice	RMCs	↑ UBL4A	↑ NF-κB signaling	Up-regulation of MiR-34b-3p reduced inflammatory response and AKI in sepsis mice	(232)
miR-21-3p	↑	_patients with sepsis, C57BL/6 mice	_	↓ SORBS2	_	Downregulation of miR-21-3p induced mitochondria ultrastructural damage and autophagy in LPS-treated mice. Levels of miR-21-3p increased in patients with cardiac dysfunction than without cardiac dysfunction.	(233)
miR-199a-5p	↑	C57BL/6 mice	HEK-293T	↓ SP-D	↑ NF-κB signaling	Down regulation of miR-199a-5p reduced D-lactic acid, DAO, FD-40, oxidative damage and inflammation.	(234)
miR-17	↓	mice	BMSCs, RAW264.7	↑ BDR4, ↑ EZH2, ↑ TRAIL	_	MiR-17 carried by BMSC-EVs reduced inflammation and apoptosis.	(235)
miR-125b	↑	120 patients with sepsis and 120 HCs	_	_	_	AUC of miR-125b: 0.658 MiR-125b was positively associated with APACHE II score, SOFA score, Scr, CRP, PCT, TNF-α, and IL-6 levels. miR-125b Was found to be an independent risk factor for mortality risk.	(236)
miR-30e	↓	septic rats	_	↑ FOSL2	↑ JAK/STAT signaling	Up-regulation of miR-30e increased proliferation and reduced apoptosis.	(237)
miR-20b-5p	↑	SD rats	HEK-293T	↓ circDMNT3B	_	Downregulation of miR-20b-5p reduced level of d-lactic acid, FD-40, MDA, diamine oxidase, IL-10, IL-6, oxidative damage and inflammatory factors level.	(238)
miR-146b	↓	CLP mice	_	↑ Notch1	_	Up-regulation of miR-146b reduced apoptosis and inflammatory response.	(239)
miR-25	↓	SD rats	H9C2	↑ PTEN, ↑ TLR4	↑ NF-κB signaling	Up-regulation of miR-25 reduced apoptosis and enhanced survival rate.	(240)
miR-21 and miR-181b	↑	septic mice	MDSCs, Gr1+CD11b + cells	↑ C/EBPβ, ↑ Stat3	_	Stat3 and C/EBPβ increased miR-21 and miR-181b expression by binding to their promoters during sepsis.	(241)
miR-17-5p	↓	septic mice	LPS-induced macrophages	↑ TLR4	_	Sch B increased miR-17-5p expression and reduced inflammation.	(242)
miR-200a-3p	↑	male C57BL/6J mice	HBMECs	↑ NLRP3, ↓ Keap1, ↓ Nrf2, ↓ HO-1	_	Up-regulation of miR-200a-3p induced inflammatory response in sepsis-induced brain injury.	(243)
miR-26b	↓	14 patients with sepsis and 7 patients with septic shock and 21 HCs	MEG-01	↑ SELP, ↓ Dicer1	_	Low levels of miR-26b was correlated with the severity and mortality of sepsis.	(244)
miR-96-5p	↓	_	RAW264.7	↑ NAMPT	↑ NF-κB pathway	Up-regulation of miR-96-5p reduced inflammatory response.	(245)
miR-27a	↑	septic mice	_	_	↑ NF-κB pathway	Downregulation of miR-27a reduced inflammatory response and promoted survival of septic mice.	(246)
miR-21a-3p	↑	specific pathogen-free SD rats	NRK52E	↑ Ago2, ↑ Nrp-1	_	miR-21a-3p was found to be internalized by TECs via Nrp-1 and Ago2.	(247)
miR-574-5p	↑	118 patients with sepsis	_	_	_	miR-574-5p was associated with the death of sepsis patients.	(248)
miR-181b	↓	26 patients with sepsis, 36 patients with sepsis plus sepsis/ARDS and 16 HCs, male C57BL/6 mice	THP-1, HUVECs	↑ importin-α3	↑ NF-κB signaling pathway	Up-regulation of miR-181b reduced mortality rate, inflammation response, LPS-induced EC activation, leukocyte accumulation.	(249)
miR-182-5p	↑	pneumonia mice models	_	_	_	Downregulation of miR-182-5p reduced apoptosis, inflammation response and promoted viability and proliferation.	(250)
miR-195	↑	C57BL/6 mice	endothelial cells	↓ BCL-2, ↓ Sirt1, ↓ Pim-1	_	Downregulation of miR-182-5p reduced apoptosis, and improved survival.	(251)
miR-205	↓	male SD rats	_	_	↑ HMGB1-PTEN signaling pathway	Up-regulation of miR-205 reduced apoptosis and renal injury.	(252)
miR-21-3p	↑ in AKI group	49 patients with sepsis-induced AKI and 93 sepsis patients with non-AKI	_	↑ Scr, ↑ Cys-C, ↑ KIM-1	_	Levels of miR-21-3p was positively associated with Scr, Cys-C, and KIM-1 in the AKI group.	(253)
miR-181a-2-3p	↓	GSE46955 data set, CLP mouse model	TCMK-1	↑ GJB2	_	Up-regulation of miR-181a-2-3p reduced apoptosis and inflammatory response.	(254)
miR-21	↓	female Wistar rats	HK-2	↑ PTEN, ↓ PI3K, ↓ AKT	_	Up-regulation of miR-21 suppressed apoptosis and kidney injury.	(255)
miR-146a	↓	female ICR mice	Raw264.7	↑ JMJD3, NF-κB p65	_	GSKJ4 reduced inflammatory response by increasing miR-146a levels. Transcription of miR-146a was negatively regulated by JMJD3 through epigenetic mechanism.	(256)
miR-294	_	_	RAW264.7	TREM-1	_	miR-294 reduced TNF-α and IL-6 secretion.	(257)
miR-128-3p	↑	CLP mouse model	TCMK-1	↓ NRP1	_	Up-regulation of miR-128-3p promoted apoptosis and inflammatory response and reduced viability.	(258)
miR-146a	↓	_	H9C2	↓ ErbB4, ↑ TRAF6, ↑ IRAK1	_	Up-regulation of miR-146a reduced apoptosis and inflammatory response and promoted viability.	(259)
miR-511	↑ in S mice	C57BL/6J (B) mice, SPRET/Ei (S) mice,	_	Low protein expression of TNFR1 in S mice	_	miR-511 was induced by glucocorticoids. miR-511 inhibited endotoxemia and experimental hepatitis.	(260)
miR-376b	↓ in sepsis with AKI group	20 Patients with sepsis with AKI, 20 patients with sepsis without AKI and 10 HCs, male C57BL/6 mice	BUMPT	NF-κB, NFKBIZ	_	miR-376b inhibited NF-κB inhibitor ζ (NFKBIZ) expression and NF-κB inhibited miR-376b expression so they created a negative feedback loop.	(261)
miR-155	↑	female BALB/c mice	_	_	_	DXM treatment suppressed the expression of miRNA-155.	(262)
miR-133a	↑	223 patients with sepsis and 76 HCs, C57Bl/6 mice	_	_	_	High levels of miR-133a was correlated with disease severity, inflammatory response, bacterial infection, and organ failure and predicted an unfavorable outcome of patients.	(263)
miR-203	↓	clean grade Kunming mice	HEK-293T	↑ VNN1	↓ AKT signaling pathway	Up-regulation of miR-203 reduced apoptosis, inflammatory response, MDA, ALT, and AST in lung tissues, PMN and PAM levels in BALF and increased SOD activity.	(264)
miR-223	↑	187 patients with sepsis and 186 HCs	_	_	_	AUC for miR-223: 0.754, Plasma miR-223 was associated with disease severity and inflammatory factor levels. miR-223 was found to predict sepsis risk independently.	(265)
miR-146a	↓	patients with sepsis and HCs	Human primary T cells	↑ PRKCϵ	_	Reduced levels of miR-146a contributes to the pathogenesis of sepsis.	(266)
miR-146-a	↓	55 patients with sepsis and 60 HCs	_	_	_	AUC for miR-146-a: 0.803 Serum levels of miR-146-a was negatively correlated with C-reactive protein, pro-calcitonin, IL-6 and TNF-α.	(267)
miR-34a	↑	CLP-induced suckling rats	U937	_	↑ STAT3 pathway	Up-regulation of miR-34a promoted iNOS secretion from pulmonary macrophages.	(268)
hsa-miR-346	↓	_	RAW264.7	↑ lncRNA MALAT1, ↑ SMAD3	_	Up-regulation of hsa-miR-346 promoted proliferation.	(269)
miR-214	↓	male Kunming mice	_	↑ PTEN	↓ AKT/mTOR pathway	Up-regulation of miR-214 reduced oxidative stress and autophagy, so ameliorated CLP-induced AKI.	(270)
miR-27a	↑	LPS induced sepsis mice model	H9C2	↓ rhTNFR:Fc, ↓ Nrf2	_	rhTNFR:Fc elevated viability and reduced apoptosis by increasing Nrf2 levels and reducing miR-27a levels.	(271)
miR-150	↓ in non-survival group	48 patients with septic shock (23 survival patients and 25 non-survival patients)	_	_	_	MiR-150 level was positively associated with cardiac index and negatively with EVLWI and PVPI.	(272)
miR-148a-3p	↑	male adult wild-type mice and myeloid-specific RBP-J-deficient mice	RAW264.7	_	Notch signaling and NF-κB pathway	Up-regulation of miR-148a-3p increased proinflammatory cytokines and decreased protective effect of EVs in LPS induced sepsis.	(273)
miR-218-5p	↑	male ICR mice	GMCs	↓ HO-1	_	miR-218-5p was reduced in honokiol-treated septic mice, so the survival rate was increased.	(274)
miR-425-5p	↓	C57BL/6 mice	hepatocytes	↑ RIP1	_	Up-regulation of miR-425-5p reduced inflammatory response and sepsis-related liver damage.	(275)
miR-122	↑ in CA group	168 patients with sepsis (CA group and CN group)	_	_	_	Serum levels of miR-122 were associated with APTT ratios, FIB and antithrombin III levels.	(275)
miR-101-3p	↑	27 patients with SIC and 15 HCs, male SD rats	H9C2	↓ DUSP1	↑ MAPK p38 and NF-κB pathways.	Downregulation of reduced apoptosis and inflammatory response.	(276)
miR-124	↓	mouse model of ALI	_	↑ MAPK14	↑ MAPK signaling pathway	Up-regulation of miR-124 reduced apoptosis and inflammatory response and promoted proliferation.	(277)
miR-942-5p	↓	_	HK-2	↑ FOXO3	_	Up-regulation of miR-942-5p reduced apoptosis and inflammatory response and promoted viability.	(278)
miR-23a-5p	↑	SD rats	NR8383	_	_	_	(279)
miR-1298-5p	↑	_	BEAS-2B	↓ SOCS6, ↑ STAT3	_	Up-regulation of miR-1298-5p induced cell permeability and inflammatory response and reduced proliferation.	(280)
miR-290-5p	↓	male C57BL/6J mice	MPC5	↑ CCL-2	_	Propofol increased levels of miR-290-5p and decreased CCL-2 and inflammatory response.	(281)
miR-146a	↓	C57BL/6 mice	BMDMs	_	_	Rg6 increased IL-10 and miR-146a levels so inhibited inflammatory responses.	(282)
miR-223	_	C57BL/6 mice	MSCs	Sema3A, Stat3	_	WT-exosomes encased high miR-223 levels induced cardio-protection in sepsis.	(283)
miR-608	_	_	U937, HEK293T	ELANE	_	miR-608 played an important role in posttranscriptional regulation of ELANE expression and upregulation of miR-608 reduced inflammation.	(284)
miR-124	↓	BALB/c and C57BL/6 mice	RAW264.7	↓ α7nAChR, ↑ STAT3	_	miR-124 was found to be a critical mediator for the cholinergic anti-inflammatory effect.	(285)
miR-26b	↑ in AKI group	155 patients with sepsis (68 AKI and 87 non-AKI ) and 57 patients with non-infectious SIRS	_	_	_	Urinary miR-26b levels showed an elevated mortality rate and was correlated with the severity of the disease.	(286)
miR-146a	_	Rat model of SAKI	_	_	_	DEX pretreatment could increase the expression level of miR-146a and reduce oxidative stress and inflammatory responses.	(287)
miR-29a	↑ in AKI group	74 patients with AKI and 41 without AKI	_	_	_	AUC for miR-29a: 0.82 miR-29a was found to be an independent risk factor for mortality in the septic patients.	(288)
miR-10a-5p	↑ in AKI group	74 patients with AKI and 41 without AKI	_	_	_	AUC for miR-10a-5p: 0.75 miR-10a-5p was found to be an independent risk factor for mortality in the septic patients.	
miR-155	↑	septic mice	NCM460	_	↑ NF-κB signaling	Up-regulation of miR-155 increased hyperpermeability to FITC-dextran, TNF-α and IL-6 levels, and decreased ZO-1 and Occludin expression.	(289)
miR-155	↑	male C57BL/6 mice	Raw264.7, THP-1	_	↑ PI3K/AKT signalling pathways	Curcumin inhibited inflammatory responses and miR-155 expression.	(290)
miR-497	↑ in myocardial injury group	148 patients with sepsis (58 myocardial injury group and 90 non-myocardial injury group)	_	_	_	Plasma miRNA-497 was correlated with cTnI in patients with myocardial injury.	(291)
miR-497-5p	↑	GEO database, male C57BL/6 mice	BEAS-2B	↓ IL2RB	_	Downregulation of miR-497-5p reduced apoptosis and inflammatory responses.	(292)
miR-30a	↓	_	monocytes	↑ STAT1, ↑ MD-2	_	miR-30a could inhibit STAT1-MD-2 in monocytes of sepsis.	(293)
miR-150	↓	C57BL/6 mice	HUVECs	↑ NF-κB1	_	miR-150 increased survival in patients and inhibited apoptosis and inflammatory responses.	(294)
miR-146a	_	_	THP-1	RBM4, Ago2, p38	_	Up-regulation of miR-146a inhibited p38 activation and increased Ago2-RBM4 protein interaction, so reduced inflammatory responses.	(295)
miR-146a	_	C57BL/6 mice	HEK293TN, J774.1	_	_	Up-regulation of miR-146a reduced morphine mediated hyper-inflammation.	(296)
miR-27a	↓	septic mice	_	↑ TAB3	↑ NF-κB signaling pathway	Paclitaxel pretreatment increased miR-27a levels, so decreased inflammatory responses.	(297)
miR-146a	↓ in septic patients than SIRS and HCs groups	50 patients with sepsis, 30 patients with SIRS and 20 HCs	_	_	_	AUC for miR-146a: 0.858	(298)
miR-223	↓ in septic patients than SIRS and HCs groups	50 patients with sepsis, 30 patients with SIRS and 20 HCs	_	_	_	AUC for miR-223: 0.804	
miR-339-5p	↓	septic mice	RAW264.7	↑ HMGB1, ↑ IKK-β	_	Paeonol could reduce inflammatory responses by upregulating miR-339-5p expression.	(299)
miR-99b	↑	male C57BL/6 J mice	RAW264.7	↓ MFG-E8	_	Spherical nucleic acid increased migration by inhibiting miR-99b.	(300)
miR-215-5p	↓	_	H9c2	↑ LRRFIP1, ↑ ILF3	_	miR-215-5p reduced inflammatory responses.	(301)
miR-15a	↑ in sepsis and SIRS than HCs	166 patients with sepsis, 32 patients with SIRS, and 24 HCs	_	_	_	miR-15a could distinguish sepsis/SIRS from HCs.	(302)
miR-16	↑ in sepsis and SIRS than HCs	166 patients with sepsis, 32 patients with SIRS, and 24 HCs	_	_	_	miR-16 could distinguish sepsis/SIRS from HCs.	

miRNAs and Sepsis. AKI, Acute kidney injury; HCs, healthy controls; AUC, significant higher area under curve; CRP, C-reactive protein; TLC, total leucocytes count; SD, Sprague-Dawley; SOFA, sequential organ failure assessment; Scr, serum creatinine; WBC, white blood cell; PCT, procalcitonin; APACHE, physiology and chronic health evaluation; CLP, cecal ligation and puncture; ERS, endoplasmic reticulum stress; AUC, area under the ROC curve; SAE, sepsis-associated encephalopathy; BUN, blood urine nitrogen; rIPC, remote ischemic preconditioning; SPF, specific pathogen-free; GEO, Gene Expression Omnibus; SIMI, sepsis-induced myocardial injury; Tregs, regulatory T-cells; Sch B, Schisandrin B; DXM, dexamethasone; MDA, malondialdehyde; ALT, aminotransferase; AST, aspartate aminotransferase; PAM; pulmonary alveolar macrophages; PMN, polymorphonuclear neutrophils; BALF, bronchoalveolar lavage fluid; SOD, superoxide dismutase; CA, coagulation abnormal; CN, coagulation normal; APTT, serum activated partial thromboplastin time; FIB, fibrinogen; SIC, sepsis-induced cardiomyopathy; SIRS, systemic inflammatory response syndrome; DEX, dexmedetomidine; SAKI, sepsis-induced acute kidney injury).

## CircRNAs and sepsis

CircRNAs are a recently appreciated group of non-coding RNAs with enclosed circular configuration formed by covalent bonds between two ends of linear transcripts. However, some of these transcripts have been shown to produce proteins. They mostly exert regulatory functions in the transcriptome. Impact of circRNAs in the sepsis has been assessed by several groups (303). For instance, circC3P1 has been shown to attenuate production of inflammatory cytokines and decrease cell apoptosis in sepsis-associated acute lung injury *via* influencing expression of miR‐21 (304).

A microarray-based has shown differential expression of 132 circRNAs between sepsis patients and healthy controls among them have been hsa_circRNA_104484 and hsa_circRNA_104670 whose up-regulation in sepsis serum exosomes has been verified been RT-PCR. Expression levels of these two circRNAs have been suggested as diagnostic biomarkers for sepsis (305).

CircVMA21 is another circRNA that has been shown to ameliorate sepsis‐related acute kidney injury through modulation of oxidative stress and inflammatory responses *via* miR‐9‐3p/SMG1 axis (306). Circ_0114428/miR-495-3p/CRBN axis is another molecular axis which is involved in the pathoetiology of sepsis‐related acute kidney injury (307). Moreover, expression levels of circPRKCI have been correlated with sepsis risk, severity of sepsis and mortality during a period of 28 days (308). **Table 3** summarizes the role of circRNAs in sepsis.

**Table 3:** CircRNAs and Sepsis.

circRNA	Pattern of Expression	Clinical Samples/Animal Model	Assessed Cell Lines	Targets / Regulators	Signaling Pathways	Description	Reference
circC3P1	↓	male C57BL/6 mice	MPVECs	↑ miR-21	_	Upregulation of circC3P1 reduced pulmonary injury, inflammatory responses and apoptosis.	(304)
hsa_circRNA_104484	↑	25 patients with sepsis and 22 HCs	_	_	_	Hsa_circRNA_104484 showed the potential to be used as diagnostic marker for sepsis.	(305)
hsa_circRNA_104670	↑	25 patients with sepsis and 22 HCs	_	_	_	Hsa_circRNA_104670 showed the potential to be used as diagnostic marker for sepsis.	
circVMA21	↓	CLP rats	HK-2, WI-38	↑ miR-9-39, ↓ SMG1	–	CircVMA21 reduced apoptosis, inflammatory responses and oxidative stress.	(306)
circ-PRKCI	↓	121 patients with sepsis and 60 HCs	_	↑ miR-545	_	Low levels of circ-PRKCI were correlated with sepsis risk, clinical disease severity and 28-day mortality risk.	(308)
circDNMT3B	↓	male SD rats	Caco2	↑ miR-20b-5p, ↓ SOD	_	Downregulation of circDNMT3B decreased cell survival and increased apoptosis, inflammatory responses and oxidative damage.	(238)
circ_0114428	↑	_	HK2	↓ miR-495-3p, ↑ CRBN	_	Downregulation of circ_0114428 decreased apoptosis, inflammatory responses, oxidative stress, and ER stress.	(307)
circ_0001105	↓	septic rats	_	↑ YAP1	_	Up-regulation of circ_0001105 decreased apoptosis, inflammatory responses and oxidative damage .	(309)
circ_Ttc3	↓	CLP rats	_	↑ miR-148a, ↓ Rcan2	_	Up-regulation of circ_Ttc3 decreased inflammatory responses and oxidative stress in AKI rats.	(310)
circPRKCI	↓	patients with sepsis and HCs	HK2	↑ miR-545, ↓ ZEB2	NF-kB pathway	Up-regulation of circPRKCI reduced LPS-induced cell injury and inflammatory responses.	(311)
circ_0003420	↑	_patients with sepsis and HCs	Kupffer cells	↓ NPAS4	_	Up-regulation of circ_0003420 increased apoptosis, inflammatory responses and decreased proliferation.	(312)
circ-Fryl	↑ in ADSC exosomes	septic mouse model	ADSCs, LPS-induced AEC damage model	miR-490-3p, ↑ SIRT3 in ADSC exosomes	SIRT3/AMPK signaling	Up-regulation of circ-Fryl increased autophagy and decreased apoptosis and inflammatory responses.	(313)
circ_0091702	↓	_	HK2	↑ miR-182, ↓ PDE7A	_	Up-regulation of circ_0091702 reduced LPS-induced cell injury.	(314)
circVMA21	↓	_	THP-1	↑ miR-199a-5p, ↓ NRP1	_	Up-regulation of circVMA21 reduced apoptosis, inflammatory responses and oxidative stress.	(315)
circTLK1	↑	wistar rats	HK-2, 293T	↓ miR-106a-5p, ↑ HMGB1	_	Downregulation of circTLK1 reduced apoptosis, inflammatory responses and oxidative stress.	(316)
circFADS2	↑	50 patients with sepsis and 50 HCs	HBEpCs	↓ mature miR-15a-5p	_	Up-regulation of circFADS2 reduced miR-15a-5p overexpression-induced apoptosis.	(317)
circ_0091702	↓	_	HK2	↑ miR-545-3p, ↓ THBS2.	_	Up-regulation of circ_0091702 reduced LPS-induced HK2 cell injury.	(318)
hsa_circ_0068,888	↓	_	HK-2	↑ miR-21-5p	_	Up-regulation of hsa_circ_0068,888 reduced inflammatory response and oxidative stress and increased viability.	(319)
circPTK2	↑	C57BL/6 mice	BV2 microglia	↓ miR-181c-5p, ↑ HMGB1	_	Downregulation of circPTK2 reduced apoptosis, inflammatory responses.	(320)
circ-FANCA	↑	19 patients with sepsis and 19 HCs	HK2	↓ miR-93-5p, ↑ OXSR1	_	Downregulation of circ-FANCA reduced apoptosis, inflammatory responses and oxidative stress and increased proliferation.	(321)
circANKRD36	↑	60 patients with sepsis-induced ARDS	RAW264.7	↓ miR-330, ↑ ROCK1	_	Downregulation of circANKRD36 reduced viability and migration and alleviated inflammatory responses.	(322)
circPRKCI	↓	_	HK2	↑ miR-106b-5p, ↓ GAB1	_	Up-regulation of circPRKCI reduced apoptosis, inflammatory responses and oxidative stress and increased viability.	(323)

HCs, healthy controls; AKI, acute kidney injury; ARDS, acute respiratory distress syndrome.

## Discussion

A vast body of literature points to the involvement of lncRNAs, miRNAs and circRNAs in the pathoetiology of sepsis-related complications. NEAT1, MALAT1, MEG3, THRIL, XIST, CRNDE, ZFAS1, HULC, MIAT and TUG1 are among lncRNAs with the strongest evidence for their participation in this process. NEAT1 as the mostly assessed lncRNA in this regard has been shown to act as a molecular sponge for let-7a, let-7b-5p, miR-370-3p, miR-124, miR-125, miR-17-5p, miR-16-5p, miR-93-5p, miR-370-3p, miR-144-3p, miR-944, miR495-3p, miR-22-3p, miR-31-5p and miR-590-3p. Through sequestering these miRNAs, NEAT1 can affect several molecular pathways in the course of sepsis. It can enhance immune responses and the related injury in target organs, thus participating in sepsis-related multiple organ damage.

Similar to lncRNAs, circRNAs influence course of sepsis mainly through acting as molecular sponges for miRNAs. circC3P1/miR-21, circVMA21/miR-9, circVMA21/miR-199a-5p, circ-PRKCI/miR-545, circPRKCI/miR-106b-5p, circDNMT3B/miR-20b-5p, circ_0114428/miR-495-3p, circ_Ttc3/miR-148a, circPRKCI/miR-454, circ-Fryl/miR-490-3p, circ_0091702/miR-182, circTLK1/miR-106a-5p, circFADS2/miR-15a-5p, circ_0091702/miR-545-3p, hsa_circ_0068,888/miR-21-5p, circPTK2/miR-181c-5p, circ-FANCA/miR-93-5p and circANKRD36/miR-330 are among circRNA/miRNA axes which are involved in the pathophysiology of sepsis-related conditions.

NF‐κB, PI3K/AKT, JAK/STAT and Wnt/β‐catenin pathways are the most important pathways being regulated by lncRNAs, circRNAs and miRNAs in the context of sepsis. These transcripts, particularly miRNAs can be used as diagnostic or prognostic markers in sepsis. Expression levels of these regulatory transcripts might be used for diagnosis of organ specific damages during the course of sepsis.

In general, the pathophysiology of sepsis is considered as an initial hyperinflammatory phase (“cytokine storm”) followed by a protracted immunosuppressive phase. Since no data is available about the differential expression of non-coding RNAs during these two distinct phases, future studies are needed to evaluate expression patterns of non-coding RNAs in these two phases. It is possible that some of the non-coding RNAs that suppress the immune response could be used as biomarkers to indicate the immunoparalysis in sepsis.

From a therapeutic point of view, several *in vitro* and *in vivo* studies have shown that up-regulation/silencing of circRNAs, lncRNAs and miRNAs can ameliorate the pathologic events in the target organs, particularly heart and kidney during sepsis. Yet, this field is still in its infancy needing verification in additional animal models and cell lines. Moreover, since sepsis is an emergency situation, any therapeutic option should be verified in terms of bioavailability, efficiency and instant amelioration of pathological events.

Since the pathoetiology of sepsis-related complications is not completely understood, high throughput sequencing strategies focusing on different classes of non-coding as well coding RNAs are necessary to find the complicated networks between these transcripts in the context of sepsis.

## Author Contributions

SG-F wrote the draft and revised it. MT designed and supervised the study. NA, BH, and TK collected the data and designed the figures and tables. All authors contributed to the article and approved the submitted version.

## Conflict of Interest

The authors declare that the research was conducted in the absence of any commercial or financial relationships that could be construed as a potential conflict of interest.

## Publisher’s Note

All claims expressed in this article are solely those of the authors and do not necessarily represent those of their affiliated organizations, or those of the publisher, the editors and the reviewers. Any product that may be evaluated in this article, or claim that may be made by its manufacturer, is not guaranteed or endorsed by the publisher.
